# A1 reactive astrocytes and a loss of TREM2 are associated with an early stage of pathology in a mouse model of cerebral amyloid angiopathy

**DOI:** 10.1186/s12974-020-01900-7

**Published:** 2020-07-25

**Authors:** Xavier Taylor, Pablo Cisternas, Yanwen You, Yingjian You, Shunian Xiang, Yamil Marambio, Jie Zhang, Ruben Vidal, Cristian A. Lasagna-Reeves

**Affiliations:** 1 Stark Neurosciences Research Institute, Indiana University School of Medicine, Neurosciences Research Building 214G, 320 West 15th Street, Indianapolis, IN 46202 USA; 2 Department of Anatomy, Cell Biology, & Physiology, Indiana University School of Medicine, Indianapolis, IN 46202 USA; 3 Department of Medical and Molecular Genetics, Indiana University School of Medicine, Indianapolis, IN 46202 USA; 4 Department of Pathology and Laboratory Medicine, Indiana University School of Medicine, Indianapolis, IN 46202 USA

**Keywords:** Cerebral amyloid angiopathy (CAA), Astrogliosis, Alzheimer’s disease (AD), Neuroinflammation, Triggering receptor expressed on myeloid cells 2 (TREM2), Vascular amyloid

## Abstract

**Background:**

Cerebral amyloid angiopathy (CAA) is typified by the cerebrovascular deposition of amyloid. The mechanisms underlying the contribution of CAA to neurodegeneration are not currently understood. Although CAA is highly associated with the accumulation of amyloid beta (Aβ), other amyloids are known to associate with the vasculature. Alzheimer’s disease (AD) is characterized by parenchymal Aβ deposition, intracellular accumulation of tau, and significant neuroinflammation. CAA increases with age and is present in 85–95% of individuals with AD. A substantial amount of research has focused on understanding the connection between parenchymal amyloid and glial activation and neuroinflammation, while associations between vascular amyloid pathology and glial reactivity remain understudied.

**Methods:**

Here, we dissect the glial and immune responses associated with early-stage CAA with histological, biochemical, and gene expression analyses in a mouse model of familial Danish dementia (FDD), a neurodegenerative disease characterized by the vascular accumulation of Danish amyloid (ADan). Findings observed in this CAA mouse model were complemented with primary culture assays.

**Results:**

We demonstrate that early-stage CAA is associated with dysregulation in immune response networks and lipid processing, severe astrogliosis with an A1 astrocytic phenotype, and decreased levels of TREM2 with no reactive microgliosis. Our results also indicate how cholesterol accumulation and ApoE are associated with vascular amyloid deposits at the early stages of pathology. We also demonstrate A1 astrocytic mediation of TREM2 and microglia homeostasis.

**Conclusion:**

The initial glial response associated with early-stage CAA is characterized by the upregulation of A1 astrocytes without significant microglial reactivity. Gene expression analysis revealed that several AD risk factors involved in immune response and lipid processing may also play a preponderant role in CAA. This study contributes to the increasing evidence that brain cholesterol metabolism, ApoE, and TREM2 signaling are major players in the pathogenesis of AD-related dementias, including CAA. Understanding the basis for possible differential effects of glial response, ApoE, and TREM2 signaling on parenchymal plaques versus vascular amyloid deposits provides important insight for developing future therapeutic interventions.

## Introduction

Alzheimer’s disease (AD) is the most common form of dementia and is characterized by the extracellular deposition of parenchymal ß-amyloid (Aß), intracellular accumulation of tau as neurofibrillary tangles (NFTs), synaptic loss, and significant inflammation [1, 2]. Cerebral amyloid angiopathy (CAA) is typified by the cerebrovascular deposition of amyloid and has a close molecular relationship with AD while remaining clinically distinct. Vascular amyloid accumulation is present in approximately 85–95% of individuals with AD [3, 4], positioning CAA as one of the strongest vascular contributors to age-related cognitive decline [5, 6]. The mechanisms responsible for CAA pathogenesis and its downstream effects on the brain are complex and incompletely understood. Despite the strong association of CAA with Aß accumulation [4], other amyloids also associate with the vasculature, suggesting that CAA is a group of biochemically and genetically diverse disorders unified by amyloid deposit accumulation in arterial blood vessel walls, and, in some cases, capillaries of the CNS parenchyma and leptomeninges [7–9].

Increasing evidence suggests a link between neuroinflammation and neuronal dysfunction in AD, orchestrated by the progressive activation of microglia and astrocytes, leading to the overproduction of pro-inflammatory molecules [10]. Microglial activation occurs early in AD pathology [11–13] with known roles in phagocytic activity; however, in the vicinity of amyloid plaques, these glial cells become pro-inflammatory, contributing to neurotoxicity in the late stages of disease [14, 15]. Astrocytes are also suggested to play a key role in AD progression and can be activated by amyloid plaques, resulting in the overexpression of cytokines and excessive oxidative stress [16]. The dysfunction between astrocytes and the neighboring neurons disrupts synaptic connections and initiates a cascade of neuronal injury [17, 18]. It has recently been shown that activation of microglia and astrocytes might not be independent events [19], and that activated microglia can directly polarize a subset of astrocytes (designated A1 astrocytes) toward a neurotoxic phenotype [19]. This astrocytic subtype is characterized by an increased expression of complement 3 (C3) [19]. A1 astrocytes have been identified in human cases and mouse models for AD, Huntington’s disease (HD), ALS, multiple sclerosis (MS), Parkinson’s disease (PD), prion disease, and frontotemporal dementia (FTD) [19–22]; however, the presence of A1 astrocytes has yet to be reported in the context of CAA.

Over 40% of genes identified as late-onset AD (LOAD) risk factors are immune-related genes predominantly expressed in microglia and astrocytes [23–25], enhancing the connection of the immune system to AD pathogenesis. Over the past decades, the molecular and cellular connections between parenchymal amyloid and glial activation and neuroinflammation have been widely studied [13, 26], while the relationship between vascular amyloid pathology and glial reactivity has mainly been reported based on neuropathological observations [27–29]. For instance, TREM2, one of the most studied AD-immune risk factors, has always been analyzed in the context of parenchymal amyloid [30–34], but no study has described its relationship with vascular amyloid pathology.

Therefore, we decided to dissect the glial responses associated with early-stage CAA in a mouse model of familial Danish dementia (FDD). FDD is characterized by the presence of CAA, consisting of the ~ 4-kDa ADan amyloid, in leptomeninges and vessels of the gray and white matter. Genetic analysis in patients with FDD revealed the presence of a 10-nucleotide duplication insertion in the 3′-end of the coding region of the BRI2 gene. This frameshift mutation generates an ADan precursor protein of 277 amino acids, of which the ~ 4-kDa ADan amyloid subunit comprises the last 34 amino acids [35]. Cotton wool-like plaques in the vicinity of blood vessels with amyloid and tau NFTs are also observed in FDD patients [36]. The mouse model for FDD (Tg-FDD) used in this study consistently exhibits CAA, primarily in leptomeningeal cerebellar vessels [37] and in the large and medium-sized parenchymal and penetrating vessels of the brain. At a late stage of CAA pathology, perivascular tau immunoreactive deposits and qualitative glial activation have also been observed in this model [38]. These observations make the Tg-FDD mice an ideal model for the study of glial reactivity and neuroimmune responses in early-stage CAA pathology.

In the present study, we show that early-stage vascular amyloid pathology in the Tg-FDD model is associated with severe astrogliosis with an A1 astrocytic phenotype without significant reactive microgliosis. We also observed that a genetic network of immune and lipid processing genes is highly dysregulated, directly impairing cholesterol, ApoE, and TREM2. Our results suggest that the lipid-ApoE-TREM2 pathway could play a preponderant role in CAA, as previously described in AD, and that in the context of vascular amyloid, astrogliosis is an early event in which activated astrocytes may directly influence microglial homeostasis.

## Materials and methods

### Transgenic mouse model

Tg-FDD and wild-type C57/BL6J (WT) (JAX stock #000664) male and female mice were used for our experiments, including cellular, biochemical, and immunohistochemistry (IHC) analyses. The Tg-FDD mouse model expresses an FDD-associated human mutant BRI2 transgene that leads to the vascular accumulation of ADan amyloid [37]. Mice were housed at the Indiana University School of Medicine (IUSM) animal care facility and were maintained according to USDA standards (12-hr light/dark cycle, food and water ad libitum), per the Guide for the Care and Use of Laboratory Animals (National Institutes of Health, Bethesda, MD). Animals were anesthetized and euthanized according to the IUSM Institutional Animal Care and Use Committee-approved procedures. Mice were deeply anesthetized prior to decapitation. After sacrifice, brains were removed and stored at − 80 °C or formalin-fixed as previously described [37]. For all described experiments, 3- or 9-month-old animals were utilized.

### Mouse brain samples preparation and immunoblot analysis

WT and Tg-FDD brains were homogenized at a 1:10 (w/vol) ratio of brain and T-PER tissue protein extraction reagent with complete protease inhibitor cocktail (Roche), then sonicated for 30 s. Samples were then centrifuged at 13,200 rpm for 15 min at 4 °C. Next, samples were run on a NuPAGE 4–12% Bis-Tris protein gel (Invitrogen) and transferred to a nitrocellulose membrane. Primary antibodies used were anti-ApoE (1:1000, AB1907, Abcam), anti-TREM2 (1:10000, AF1729, R&D), and anti-Vinculin (1:10000, V9131, Sigma). The secondary antibodies used were goat anti-mouse HRP IgG (1:1500, A16066, Invitrogen) and goat anti-rabbit HRP IgG (1:1500, PI31460, Invitrogen). Western blot (WB) quantification results are expressed as the ratio of ApoE or TREM2 normalized by the loading control Vinculin. In all cases, we considered the control group to be 100%.

### Brain sections immunofluorescence

Paraffin sections were deparaffinized in xylene and rehydrated in ethanol (EtOH) and washed with deionized water. Then, the sections were heated with a microwave oven in low pH antigen retrieval solution (eBioscience) twice for 4 min each. After washing in PBS twice for 5 min each, the sections were blocked with PBS 5% goat serum, 5% horse serum, 2% fish gel, and 0.01% Triton X-100 for 1 h at room temperature (RT). Sections were then incubated overnight at 4 °C with the following antibodies: anti-GFAP (G3893, Sigma-Aldrich), anti-IBA1 (MABN92, Millipore), anti-CD11B (1:100, AB8878, Abcam), anti-C3 (PA5-21349, ThermoFisher), anti-ADan 1699 (gift from Dr. Ruben Vidal), anti-Desmin (smooth muscle actin) (PA5-21349, Millipore), anti-ApoE (AB1907, Abcam), and anti-TREM2 (AF1729, R&D) diluted 1:100 in blocking solution. The next day, sections were quickly washed 3 times in PBS and incubated with 1:500 biotinylated horse anti-mouse antibody (BA-200, Vector) and/or biotinylated goat anti-rabbit antibody (BA-1000, Vector) for 1 hr at RT. Thirty minutes in advance, the Vectastain Elite ABC peroxidase kit (PK-6100, Vector) was prepared according to the manufacturer’s instructions. After the secondary antibody incubation, sections were incubated with the A + B solution for 30 min at RT. After 3 quick washes with PBS, tyramide dyes were prepared 1:500 in PBS, and slides were incubated with them for 10 min at RT. Slides were incubated with 3% H_2_O_2_ for 10 min at RT to stop peroxidase activity. Vascular amyloid was stained with 1% Thioflavin-S for 8 min at RT, followed by two washes in EtOH 50% and 30% for 3 min each, and a final wash in deionized water for 5 min. For cholesterol detection, sections were stained with filipin at a concentration of 250 μg/mL and incubated overnight at 4 °C, then rinsed in PBS 3 × 5 min in the dark to prevent fading. Finally, sections were washed in PBS and mounted with Vectashield mounting medium with or without DAPI (Vector Laboratories).

### Astrocytes and microglia primary cell culture

Primary glial cell cultures were prepared from newborn (postnatal day 0–3) brain cortex of C57/BL6J mice. Briefly, animals were euthanized, and their brains extracted. Brains were cut into small pieces, collected in HBSS (H9269, Sigma), and treated with 2.5% trypsin (15090-046, Gibco) and 1% DNase (EN0521, Thermo) at 37 °C for 15 min. Then, the tissue was disaggregated by pipetting, passed through a 70-μm pore cell strainer (352350, Corning), and collected in FBS. Cells were centrifuged for 5 min at 1000×*g*, and the obtained pellet was resuspended in 10 mL glial medium (Advanced DMEM/F12, 10% FBS, 100 g/mL streptomycin, 100 UI/mL penicillin, 200 μg/mL glutamine). Cells were then counted using a Luna Dual Fluorescence Cell Counter (Logos Biosystem). Cells were plated into 75-cm^2^ cell culture flask at a density of 1 × 10^6^ and incubated until 90% confluent, changing the media every 2 days. For primary microglia cell culture, confluent flasks were shaken for 1 hr at 200 rpm at 37 °C to detach microglial cells. Then, the supernatants containing microglia were collected and centrifuged for 5 min at 300×*g*. The cells were resuspended into 1 mL of glial media and counted using an automatic cell counter (Logos Biosystem) and seeded in 6-well culture plates for further experiments. The flask was treated with 0.5% trypsin (15400054, Gibco) for 10 min at 37 °C to detach the astrocytes. Then, the supernatant was collected, centrifuged at 300×*g* for 5 min, and the pellet was resuspended in glial medium. Astrocytes were quantified using an automated cell counter (Logos Biosystem) and seeded into 12-well (2 × 10^5^ cells) plates for further experiments. Primary astrocytes were treated with A1 inducing cocktail IL-1α (3 ng), TNFα (30 ng), and C1q (400 ng) for 24 h to promote A1 induction. The next day, media was replaced with fresh serum-free media and was allowed to condition for another 24 h, which we define as astrocytic conditioned media (ACM). Control-ACM or A1-ACM was then collected and lyophilized with a Speed Vac Plus SC110A and resuspended in 500 μL of PBS. The total protein concentration was determined using the Pierce BCA protein assay kit (23246, Thermo Scientific), and 50 μg of total protein was added to mouse primary microglia.

### Cell culture immunofluorescence

Astrocytes or microglia were seeded at 2 × 10^5^ cells/well in 18 mm diameter coverslips. Once the culture reached 90% confluence, the cells were fixed in PBS containing 4% paraformaldehyde (PFA) for 15 min. Cells were permeabilized for 5 min at RT in 0.25% Triton X-100 in PBS, washed twice with PBS, and incubated for 1 h at 37 °C in PBS containing 5% goat, 5% horse serum, and 2% fish gel (blocking solution). Cells were then incubated overnight at 4 °C in primary antibody diluted 1:100 in blocking solution with anti-IBA1 (MABN92, Millipore) and anti-TREM2 (AF1729, R&D). After incubation, cells were washed with PBS, then incubated with Alexa 488-conjugated goat anti-mouse antibody (1:100, Invitrogen) and Alexa 568-conjugated goat anti-rabbit antibody (1:100, Invitrogen) for 1 h at RT, then washed with PBS and mounted with Vectashield mounting medium with DAPI (Vector Laboratories). Samples were examined using a Nikon A1-R laser scanning confocal microscope coupled with Nikon AR software for orthogonal images of reconstructed three-dimensional views. At least 5–10 cells were analyzed from each image. Eight to ten images were used for each experiment, and three independent culture experiments were performed.

### Microscopy and image analysis

We used the ImageJ software (NIH) to create one index that represented changes in both astrocyte and microglia. The number of C3 (+), GFAP (+), or IBA1 (+) pixels was divided by the total number of pixels in the image and expressed as a GFAP (+) or IBA1 (+) area % [39] or C3 (+) GFAP (+) cells % [19]. For colocalization analysis, the ImageJ Coloc2 plugin was used for quantification and expressed as a percentage (% Colocalization Area). Microglia or astrocyte cell morphology was analyzed using IBA1 or GFAP and nuclear staining for Dapi. Cells were analyzed for the number of glial branches and junction using cross-sectional measurements of 10–30 randomly selected cells from 5 different micrographs per animal obtained from 20 μm image *z*-stacks as described [40]. The *z*-stack images for IBA1 or GFAP were obtained with a × 63 objective with a × 0.7 zoom and a 0.2-μm *z*-step. To fully analyze the cellular processes, only IBA1 (+) or GFAP (+) labeled cells in which the cell body was located toward the middle portion of the *z*-plane were selected for imaging. For every single staining, as a negative control, primary antibodies were omitted to determine background and autofluorescence (not shown). WT and Tg-FDD (3–4 animals per genotype) cerebral cortex and cerebellum were examined using a Leica DMi 8 epifluorescence microscope coupled with the LAS X program (Leica) or Nikon A1-R laser scanning confocal microscope coupled with Nikon AR software (Nikon).

### qPCR

Total RNA was isolated from mouse brains with the RNeasy Plus Universal Mini Kit (Qiagen). cDNA was prepared from 1 μg total RNA with High-Capacity cDNA reverse transcription kit (Life Technologies). All qPCRs were performed on QuantStudio 6 Flex Real-Time PCR system (Life Technologies). The mouse TREM2 relative gene expression was evaluated with the delta Ct method using Taqman probe sets (TREM2: Mm00451744_m1, GAPDH 4351309, Life Technologies) and TaqMan Universal PCR Master Mix.

### RNA sequencing

#### Library preparation and sequencing

Three mice per group were used for RNA sequencing experiments. The cerebellum was homogenized in Trizol, and total RNA was isolated by chloroform extraction as previously described [41]. The concentration and quality of total RNA samples were first assessed using an Agilent 2100 Bioanalyzer. An RNA Integrity Number (RIN) of five or higher was required to pass the quality control. Then, 500 ng of RNA per sample was used to prepare a dual-indexed strand-specific cDNA library using TruSeq Stranded mRNA Library Prep Kit (Illumina). The resulting libraries were assessed for quantity and size distribution using a Qubit and an Agilent 2100 Bioanalyzer, respectively. Two hundred picomolar pooled libraries were utilized per flowcell for clustering amplification on cBot using HiSeq 3000/4000 PE Cluster Kit and sequenced with 2 × 75 bp paired-end configuration on HiSeq4000 (Illumina) using HiSeq 3000/4000 PE SBS Kit. A Phred quality score (Q score) was used to measure the quality of sequencing. More than 97% of the sequencing reads reached Q30 (99.9% base call accuracy).

### Sequence alignment and gene counts

The sequencing data were first assessed using FastQC (Babraham Bioinformatics, Cambridge, UK) for quality control. Then, all sequenced libraries were mapped to the mm10 mouse genome using STAR RNA-Seq aligner [42] with the following parameter: “--outSAMmapqUnique 60.” The reads distribution across the genome was assessed using bamutils (from ngsutils) [43]. Uniquely, mapped sequencing reads were assigned to mm10 refSeq genes using featureCounts (from subread) [44] with the following parameters: “-s 2 -p –Q 10.” Quality control of sequencing and mapping results was summarized using MultiQC [45]. Genes with read count per million (CPM) > 0.5 in more than 3 of the samples were kept. The data were normalized using TMM (trimmed mean of M values) method. Differential expression analysis was performed using edgeR [46, 47]. False discovery rate (FDR) was computed from *p* values using the Benjamini-Hochberg procedure.

### Genomic pathway analysis

Ingenuity Pathway Analysis (IPA) core analysis was used to identify the perturbed gene networks in 9-month-old Tg-FDD mice compared to WT mice. The differential expression (DE) cutoff we used in the analysis is fold change greater than 1.2 and adjusted *p* value lower than 0.05. In the network figure generated by IPA, the genes marked by red color are upregulated while the genes marked by green are downregulated, and the main biological function of the network was provided. Then, for a specific perturbed gene network of interest, a heatmap showing differential expression of genes was generated to visualize the expression perturbation of genes contained in the network. Genes of interest were cross-referenced with the Agora open access portal. Agora hosts evidence for whether or not genes are associated with Alzheimer’s disease (AD). Agora also contains a list of over 500 nascent drug targets for AD that were nominated by AD researchers. The list of nominated targets was contributed by researchers from the National Institute on Aging’s Accelerating Medicines Partnership in Alzheimer’s Disease (AMP-AD) consortium as well as other research teams. Other evidence presented in Agora was either generated by AMP-AD research teams or is aggregated from publicly available data sources [48, 49].

### Statistical analyses

Experimental analysis and data collection were performed blind unless otherwise stated. *P* values were determined using the appropriate statistical method via GraphPad Prism as described. Statistical comparisons were made using a two-tailed unpaired Student’s *t* test. Data are presented as mean ± SEM unless otherwise stated. *, **, ***, and **** denote *p* < 0.05, *p* < 0.01, *p* < 0.001, and *p* < 0.0001, respectively.

### Data availability

The accession number for the RNA-Seq data reported in this paper is Gene Expression Omnibus (GEO): GSE150394.

## Results

### Reactive astrogliosis in early-stage vascular amyloid pathology in Tg-FDD mice

**Fig. 1:**
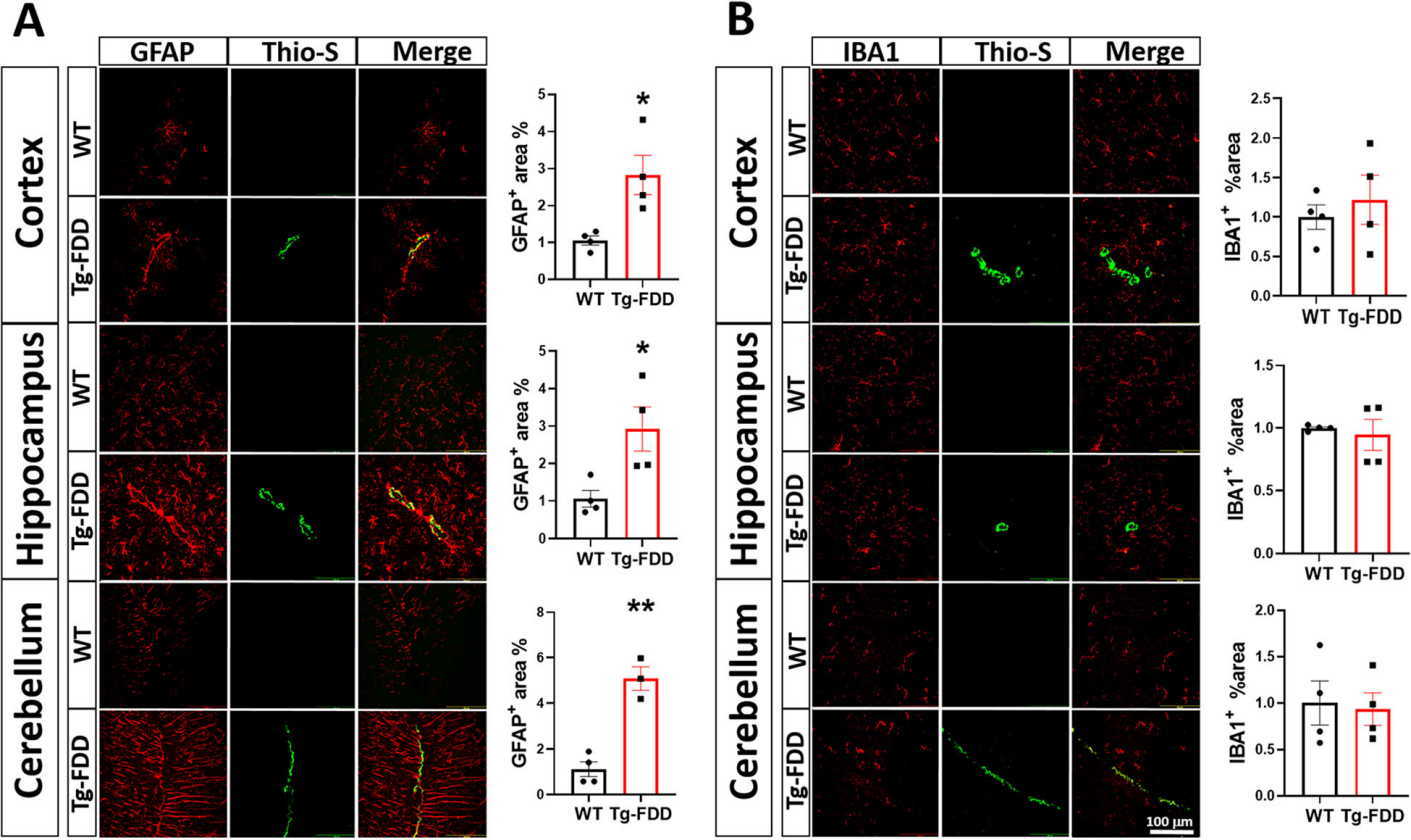
Glial immunoreactive changes in Tg-FDD mice. **a** Double-stained immunofluorescent images of amyloid (Thio-S, green) and astrocytes (GFAP, red). Quantification of GFAP^+^ area (%) of WT or Tg-FDD mice. **b** Double-stained immunofluorescent images of amyloid (Thio-S, green) and microglia (IBA1, red). Quantification of IBA1^+^ area (%) of WT or Tg-FDD mice. All are representative images of 9-month-old mice. Results are shown as the mean ± SEM of *n* = 3–4, where *p < 0.05 and **p < 0.01 as determined by unpaired Student’s *t* test. Scale bar 100 μm

Reactive gliosis is a hallmark of many human neurodegenerative diseases, including AD [50]; however, investigations into glial activation driving neuroinflammation and neurodegeneration have predominantly focused on parenchymal amyloid accumulation, with the contribution of vascular amyloid accumulation remaining understudied. To dissect glial responses associated with early-stage CAA in detail, we performed histological analyses of 9-month-old Tg-FDD mice as this age represents early-stage vascular amyloid accumulation [37]. Thio-S staining of brain sections from Tg-FDD mice revealed the presence of vascular amyloid deposits in the cortex, hippocampus, and cerebellum (Sup. Fig. 1) as previously reported [37]. Since changes in area coverage by astrocytes or microglia is a highly reliable measure of glial reactivity [51, 52], we stained brain sections for GFAP (astrocytes) and IBA1 (microglia) markers and quantified area coverage of each in the cortex, hippocampus, and cerebellum of WT and Tg-FDD mice. We observed severe astrogliosis with robust GFAP staining in all three brain regions in Tg-FDD mice in comparison with WT mice. Surprisingly, no significant change in microglial immunoreactivity was observed (Fig. 1a, b) The absence of microglial reactivity was confirmed with CD11B staining (Sup. Fig. 2). Triple staining for Thio-S, GFAP, and α-SMA (smooth muscle actin), a vascular cell marker [53], revealed that astrogliosis is accentuated in perivascular regions in 9-month-old Tg-FDD mice in comparison with WT mice (Sup. Fig. 3). Immunofluorescence for glial markers in brains from 3-month-old Tg-FDD mice, an age when no vascular amyloid is observed [37], revealed no astrogliosis (Sup. Fig. 4). This suggests that astrocytic reactivity is a response to CAA pathology, specifically, in this mouse model.

**Fig. 2:**
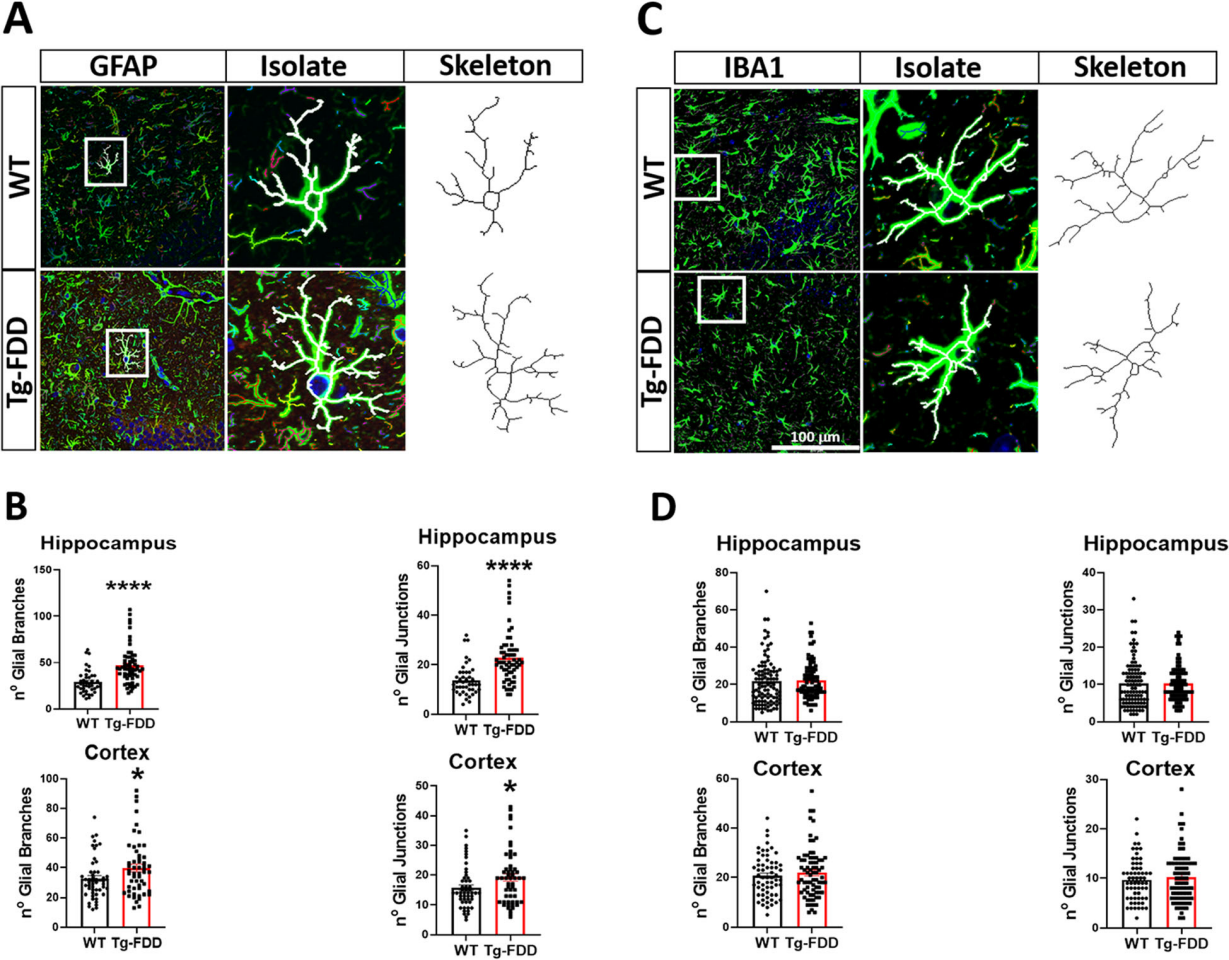
Glial morphometric changes in Tg-FDD mice. **a** Individual astrocytes (GFAP, green) were selected, isolated, thresholded, and skeletonized for analysis from brain sections of WT or Tg-FDD mice. **b** Quantification of individual astrocyte number of branches and junctions from the hippocampus and cortex. **c** Individual microglia (GFAP, green) were selected, isolated, thresholded, and skeletonized for analysis within cortex and hippocampus in brain sections of WT or Tg-FDD mice **d** Quantification of individual microglia number of branches and junctions from the hippocampus and cortex. **a** and **c** are representative images of the hippocampus of 9-month-old mice. Results are shown as the mean ± SEM of *n* = 4 per genotype and 10–30 astrocytes or microglia per animal, where *p < 0.05 and ****p < 0.0001 as determined by unpaired Student’s *t* test. Scale bar 100 μm

As glial morphology, function, and reactivity are closely related [54], we performed morphometric analyses to assess changes in individual astrocytes or microglia. In Tg-FDD mice, there is a significant increase in the number of astrocytic branches and junctions in comparison with WT (Fig. 2a, b); however, no significant changes were observed in microglia morphology (Fig. 2c, d). Overall, these results indicate that reactive astrogliosis is an early event that occurs without significant microgliosis in the pathogenesis of CAA in the Tg-FDD model.

### A1 astrocytes are highly abundant in Tg-FDD mice

**Fig. 3:**
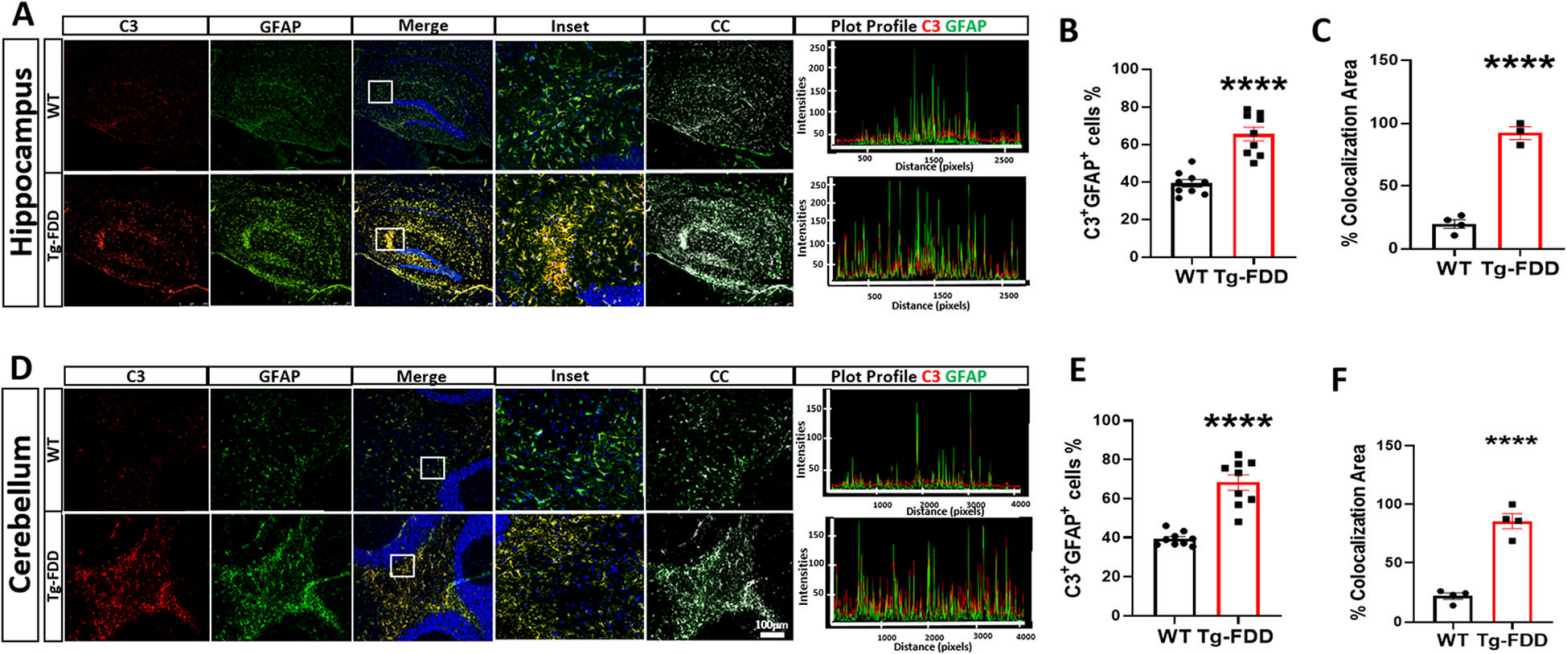
A1 Astrocytes are highly abundant in Tg-FDD. **a** Double-stained immunofluorescent images of complement component 3 (C3, red) and astrocytes (GFAP, green) in the hippocampus of WT and Tg-FDD mice. C3 and GFAP immunoreactivity overlay (Merge). **b** Quantification of C3^+^GFAP^+^ cell % from 3 regions per animal of the hippocampus of 4 WT and 3 Tg-FDD mice. **c** Quantification of % colocalization area from the hippocampus of 4 WT and 3 Tg-FDD mice. **d** Double-stained immunofluorescent images of complement component 3 (C3, red) and astrocytes (GFAP, green) in the cerebellum of WT and Tg-FDD mice. C3 and GFAP immunoreactivity overlay (Merge). **e** Quantification of C3^+^GFAP^+^ cells % from 3 regions per animal of the cerebellum of 4 WT and 3 Tg-FDD mice. **f** Quantification of % colocalization area from the cerebellum of 4 WT and 3 Tg-FDD mice. **a** and **d** are representative images of the brain regions of 9-month-old mice. Colocalization analysis (CC) was performed to determine pixel intensity correlation between C3 and GFAP signals. White pixels indicate colocalization between C3 and GFAP signal. Plot profiles of representative C3 and GFAP intensities show higher overlapping of C3 with GFAP in Tg-FDD mice in comparison with WT mice, indicating an increase of C3 positive astrocytes. Results are shown as the mean ± SEM of *n* = 3–4. Asterisks indicate significant differences, where **** *p* < 0.0001 by unpaired Student’s t test. Scale bar 100 μm

A1 Astrocytes are a newly identified astrocyte subclass triggered in response to injury, disease, and inflammatory factors. These astrocytes contribute to neuronal death in AD, Huntington’s disease (HD), ALS, multiple sclerosis (MS), Parkinson’s disease (PD), prion disease, and frontotemporal dementia (FTD) [19–22]. To determine if A1 astrocytes are present in the context of CAA, we immunostained brain sections with C3, a characteristic and significantly upregulated gene used as a specific marker of A1 astrocytes [19]. C3^+^ astrocytes were present in both WT and Tg-FDD mice; however, there was an increase of C3^+^ astrocytes in Tg-FDD mice in both the hippocampus and cerebellum (Fig. 3). Interestingly, when the intensity plot profiles of C3 and GFAP were analyzed, there was a larger overlap of both markers in Tg-FDD mice in both of these brain regions, indicating a major presence of C3^+^ astrocytes in these animals (Fig. 3a, d). Additionally, we analyzed the percentage of individual GFAP^+^ cells that were also C3^+^ in both hippocampus and cerebellum. We found that in the hippocampus, 65% of GFAP^+^ cells were also C3^+^ in Tg-FDD mice, compared with 39% in WT mice (Fig. 3b). In the cerebellum, 70% of GFAP^+^ cells were C3^+^ in Tg-FDD mice compared with 39% in WT mice (Fig. 3e). When analyzing the percent colocalization area of C3^+^ GFAP^+^ cells, 85% corresponded to C3^+^GFAP^+^ cell area in Tg-FDD mice compared with 22% in WT in the hippocampus (Fig. 3c), and 92% in Tg-FDD mice versus 20% in WT from the cerebellum (Fig. 3f). Overall, these observations indicate a strong presence of A1 astrocytes during the early stages of CAA pathology in Tg-FDD mice.

### Dysregulation of immune response and lipid processing networks associated with CAA

**Fig. 4:**
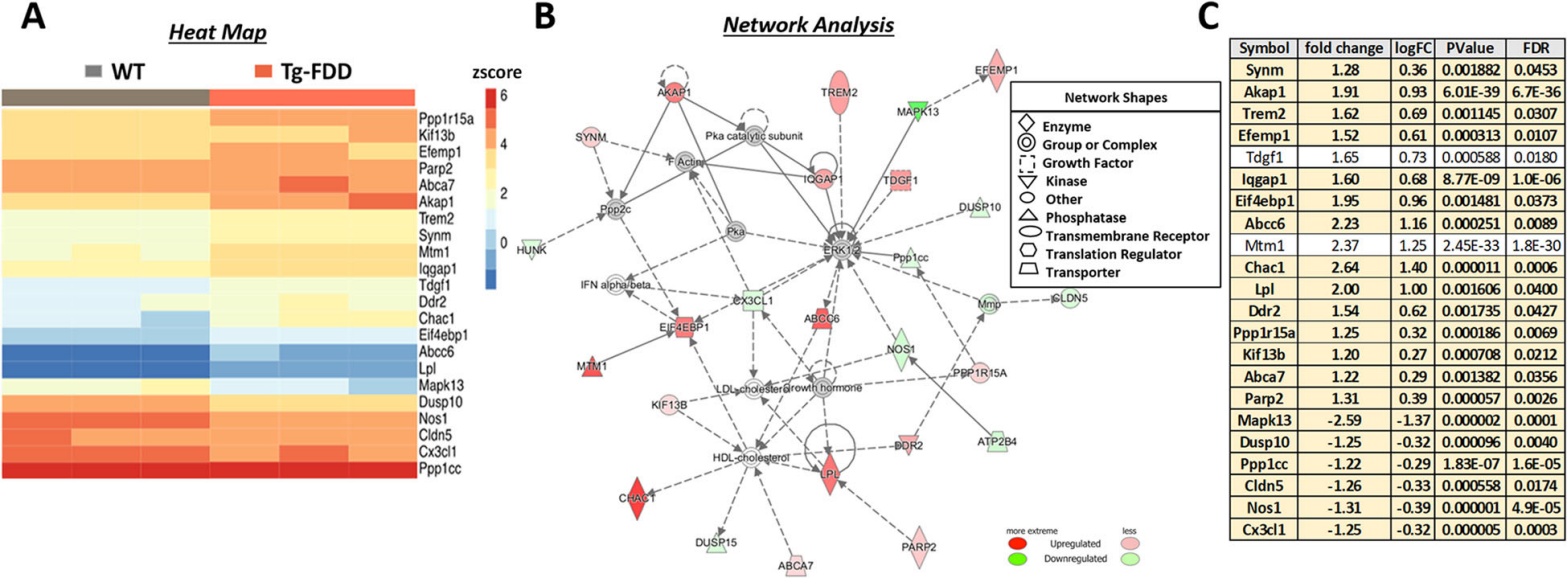
Dysregulation of immune response and lipid processing network in Tg-FDD mice. **a** Heatmap of gene expression (red = upregulation, blue = downregulation) and **b** network analysis of significantly affected genes determined by RNA-Seq in WT versus Tg-FDD mice (*n* = 3; 9-month-old). **c** Bolded genes from lipid processing and immune response network indicate genetic association with LOAD or if the gene has been nominated as a potential target for AD. logFC, log2 (fold change); FDR, false discovery rate

To analyze global transcriptional changes in response to CAA, we performed RNA-Seq analysis on 9-month-old WT and Tg-FDD mice. We used IPA core analysis to identify perturbed gene networks in Tg-FDD mice compared with WT. Remarkably, one of the top gene networks identified in the Tg-FDD model was associated with immune response and lipid processing genes (Fig. 4). Noteworthy, genetic analysis has previously implicated these genes as AD risk factors [55, 56]. For instance, many of the dysregulated genes identified in this network, such as TREM2, LPL, ABCA7, and CX3CL1 (Fig. 4), have been linked to AD pathogenesis [57–64]. To further determine whether or not other genes dysregulated in this network are associated with AD, we cross-referenced these genes with a list of nominated AD targets contributed by researchers from the National Institute on Aging’s Accelerating Medicines Partnership in Alzheimer’s Disease (AMP-AD) consortium [48, 49]. The AMP-AD has used a wide variety of patient populations and -omics strategies (genomics, transcriptomics, proteomics, metabolomics, etc.) to identify novel targets for late-onset AD therapeutic development. Our cross-reference analysis revealed that over 90% of the dysregulated genes from the immune-lipid network identified in the Tg-FDD have been indicated to have a genetic association with LOAD (bolded genes in Fig. 4c and Sup. Information 1). Overall, the RNA-Seq analysis from Tg-FDD mice indicates that vascular amyloid pathology induces significant transcriptional dysregulation of immune response and lipid processing genes with strong genetic associations to LOAD, suggesting that some known AD genetic risk factors could play a preponderant role in CAA pathogenesis.

**Fig. 5:**
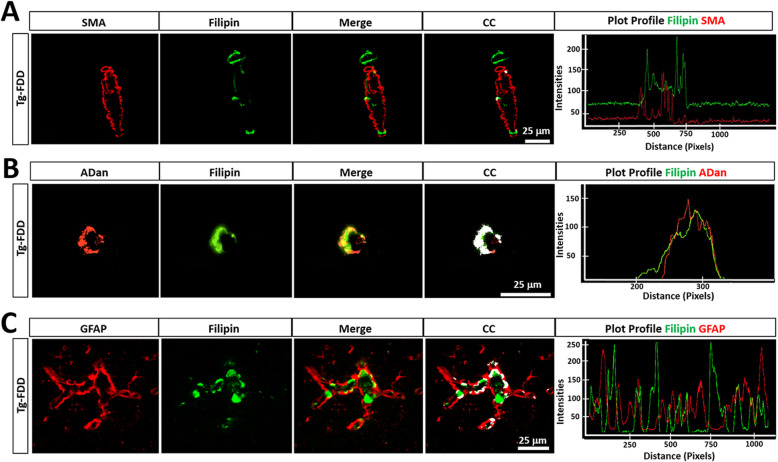
Cholesterol accumulation is associated to vascular amyloid in Tg-FDD mice. **a** Double immunofluorescence of smooth muscle actin (SMA, red) and cholesterol fFilipin, green) in Tg-FDD mice show filipin^+^ staining without association to SMA (Merge). Colocalization analysis (CC) was performed to determine pixel intensity correlation between filipin and SMA. White pixels indicate colocalization between filipin and SMA signal. Plot profiles of representative intensities showing almost no overlapping intensities of cholesterol (filipin, green) and SMA (red). **b** Double immunofluorescence of Danish amyloid (ADan, red) and cholesterol (filipin, green) in Tg-FDD mice show strong filipin^+^ colocalization and overlapping intensities (Merge). Colocalization analysis confirmed major association between filipin and vascular amyloid. White pixels indicate colocalization between filipin and ADan (red) signal. Plot profiles of representative intensities showing major overlapping intensities of cholesterol (filipin, green) and ADan (red). **c** Double immunofluorescence of astrocytes (GFAP, red) and cholesterol (filipin, green) in Tg-FDD mice show filipin^+^ show a high degree of cellular association (Merge). White pixels in CC indicate colocalization between filipin and GFAP (red) signal. Plot profiles of representative intensities showing minor overlapping intensities of cholesterol (filipin, green) and GFAP (red). Scale bar 25 μm

Considering the perturbations of lipid processing genes in Tg-FDD mice suggested by the RNA-Seq analysis, we investigated cholesterol homeostasis in response to CAA. Cholesterol is highly enriched in the brain [65], and its accumulation with parenchymal amyloid plaques has previously been reported in AD patients and in the brains of transgenic APP mice [66]; however, it has yet to be investigated in the context of vascular amyloid accumulation. To investigate cholesterol association with CAA, we performed filipin staining, a polyene macrolide antibiotic that binds to cholesterol [67]. Interestingly, we observed deposits of cholesterol accumulation in 9-month-old Tg-FDD but not in WT age match controls nor in 3-month-old Tg-FDD mice (Sup. Fig. 5). These cholesterol deposits were observed in regions of vascular amyloid accumulation in this model (Sup. Fig. 1): the cortex, hippocampus, and cerebellum (Sup. Fig. 5). To determine if this accumulation of cholesterol was within the vasculature, we performed double staining for filipin and α-SMA. No direct colocalization was observed between filipin staining and smooth muscle cells (Fig. 5a), suggesting that cholesterol does not accumulate in these cells, but in other structures or areas of the vasculature. We then analyzed a possible spatial relationship between cholesterol accumulation and vascular amyloid. Double staining revealed a strong colocalization of cholesterol with vascular amyloid deposits (Fig. 5b). To determine if cholesterol also accumulates in astrocytes in the Tg-FDD model, double staining with GFAP and filipin was performed. Our results show colocalization between GFAP and filipin only in vascular astrocytes (Fig. 5c), suggesting that this may result from an association between reactive astrocytes, vascular amyloid, and cholesterol deposits, rather than cholesterol accumulation within the astrocytes. Three-dimensional confocal views confirmed that cholesterol accumulation is indeed surrounded by activated astrocytes rather than internalized by astrocytes (Sup. Fig. 6). The colocalization between cholesterol and vascular amyloid was observed not only in the cortex (Fig. 5) but also in the hippocampus and cerebellum. These results demonstrate that cholesterol accumulation is highly associated with vascular amyloid deposits in CAA, as previously reported for parenchymal amyloid in AD [66].

**Fig. 6:**
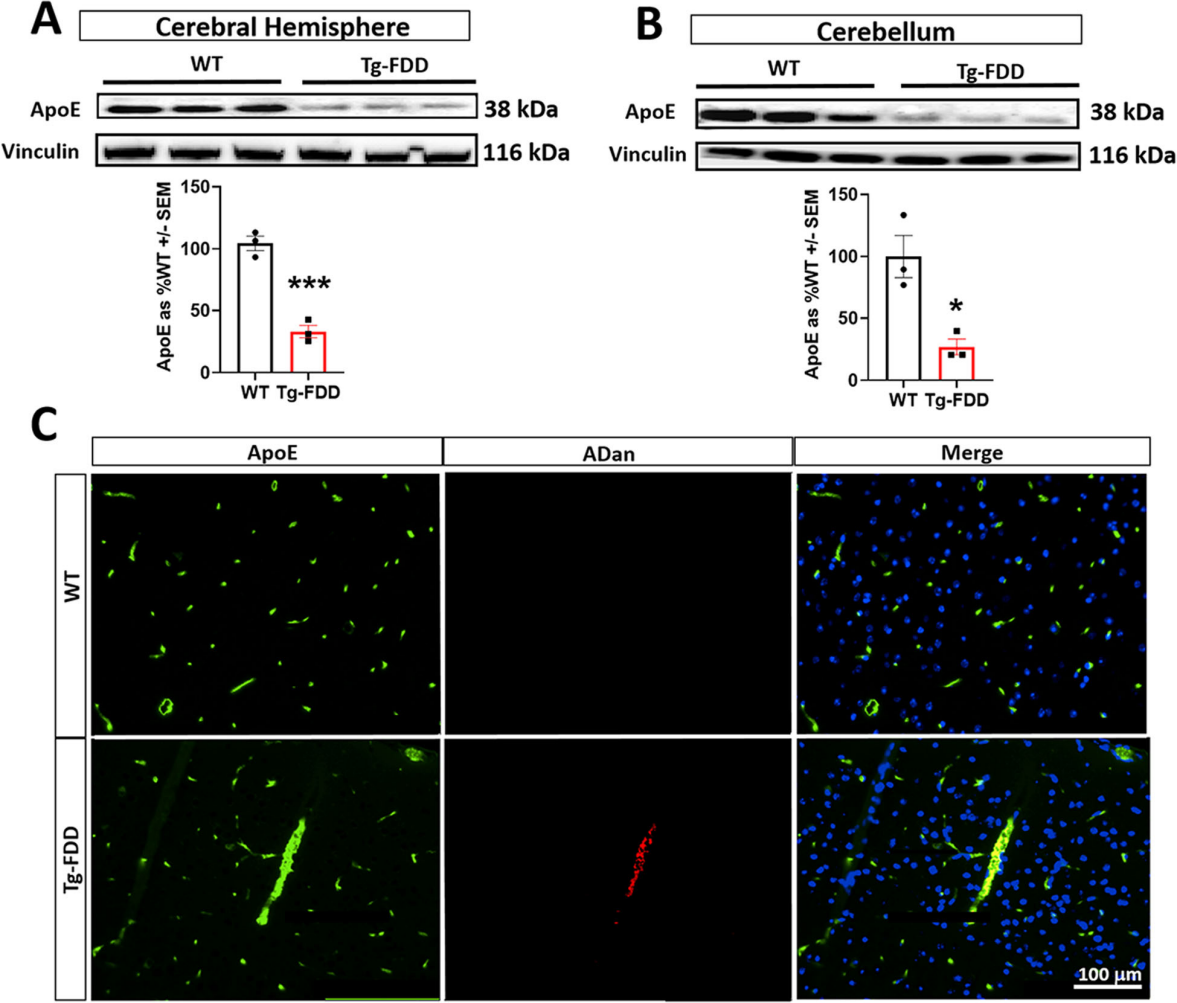
ApoE is associated with vascular amyloid in Tg-FDD mice. **a**, **b** WB analysis and quantification of ApoE from the cerebral hemisphere and cerebellum of WT and Tg-FDD mice. Vinculin was used as a loading control. For quantifications, error bars represented ± SEM *n* = 3, where *p < 0.05 of ***p < 0.001, unpaired Student’s *t* test. **c** Double immunofluorescence images of apolipoprotein E (ApoE, green) and Danish amyloid (ADan, red) in WT and Tg-FDD brain sections show a high degree of association of ApoE with vascular amyloid in the Tg-FDD (merge). All are representative images of 9-month-old mice cortex. Scale bar 100 μm

As apolipoprotein-E (ApoE) is implicated in regulating vascular integrity, cholesterol transport, and immune responses, and ApoE_4_ allele is the strongest genetic risk factor for both AD and CAA [68–72], we evaluated ApoE in Tg-FDD mice. The RNA-Seq analysis did not reveal changes in ApoE mRNA levels between WT and Tg-FDD mice. However, ApoE protein levels were significantly decreased in the Tg-FDD in comparison with WT mice (Fig. 6a, b). It has been previously reported that ApoE accumulates in the brain vasculature in the Tg-FDD model [37]. Our results confirm this and show that ApoE colocalizes with vascular amyloid deposits (Fig. 6c), suggesting that the decrease in ApoE protein levels (Fig. 6a, b) may be due to a decrease in its solubility resulting from its association with insoluble vascular amyloid deposits.

**Fig. 7:**
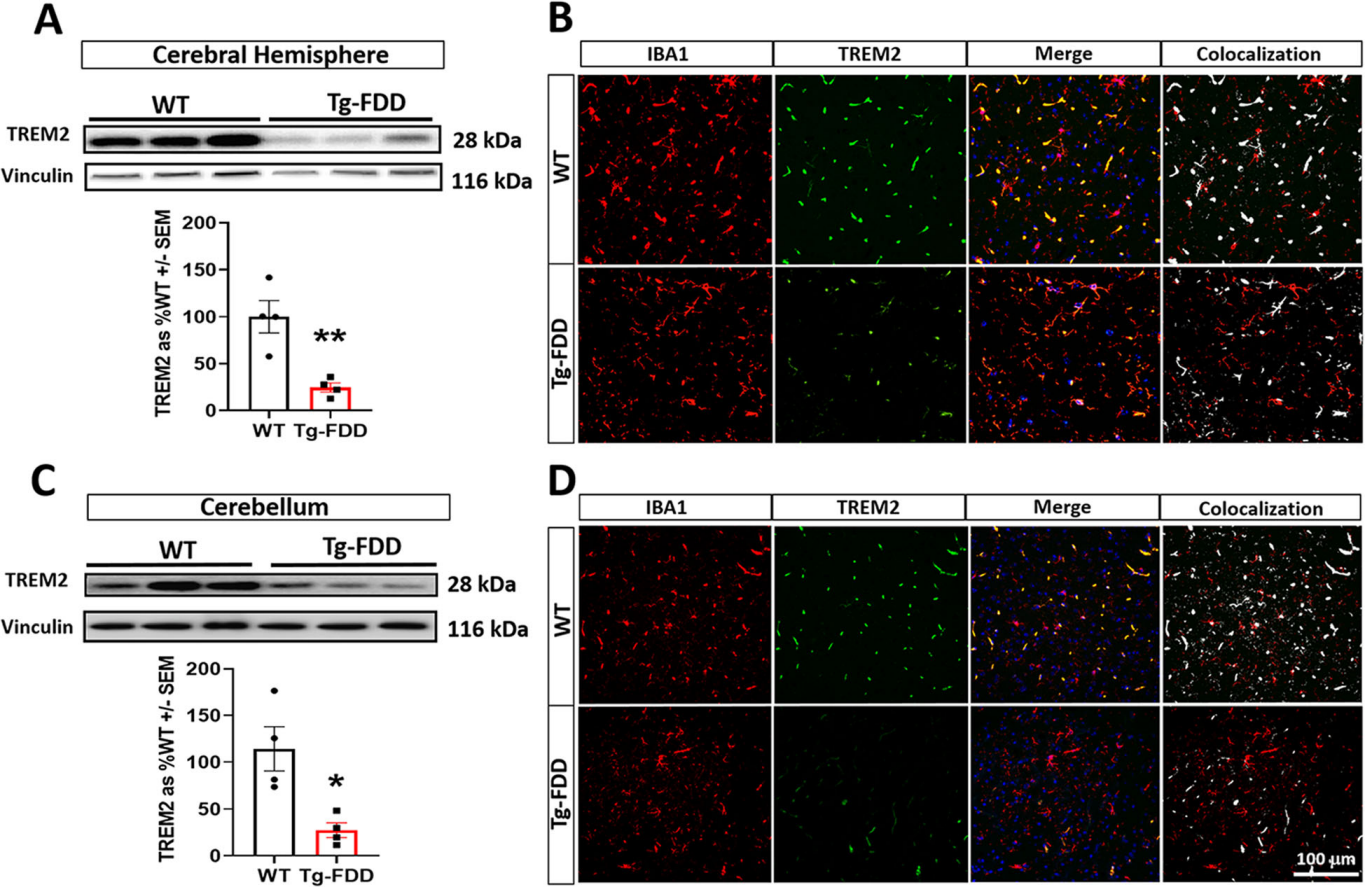
Decreased TREM2 protein levels in Tg-FDD mice microglia. **a** WB analysis and quantification of TREM2 protein levels in the cerebral hemisphere of WT and Tg-FDD mice. **b** Double immunofluorescence images of microglia (IBA1, red) and TREM2 (green) in the hippocampus of WT and Tg-FDD mice. **c** WB analysis and quantification of TREM2 protein levels in the cerebellum of WT and Tg-FDD mice. **d** Double immunofluorescence images of microglia (IBA1, red) and TREM2 (green) in the cerebellum of WT and Tg-FDD mice. For WB analysis (**a** and **c**), TREM2 levels were normalized by vinculin. For WB quantifications, error bars represented ± SEM *n* = 4, where *p < 0.05 of ***p < 0.001, unpaired Student’s *t* test. For double immunofluorescence (**b** and **d**), a decrease of TREM2 (green) positive microglia (IBA1, red) was observed in WT versus Tg-FDD mice in both brain regions (Merge). Colocalization analysis (CC) was performed to determine pixel intensity correlation between TREM2 and IBA1. White pixels indicate colocalization between TREM2 and IBA1 signal. All are representative images of 9-month-old WT or Tg-FDD mice. Scale bar 100 μm

Through the RNA-Seq analysis, we identified TREM2 as one of the upregulated immune-related genes in the Tg-FDD mouse (Fig. 4). Since TREM2 plays a critical role in immune regulation and AD pathogenesis [73], we further characterized this microglial receptor in the context of CAA. We first confirmed the increases in TREM2 mRNA levels in the Tg-FDD model by qPCR (Sup. Fig. 7). Next, we performed WB to detect the expression of TREM2 protein. Interestingly, protein levels of TREM2 were significantly decreased in 9-month-old Tg-FDD mice compared with WT mice (Fig. 7a, c). Additionally, we performed double staining for IBA1 and TREM2 in WT and Tg-FDD mice. Strikingly, we observed a significant decrease of TREM2-positive microglia in the Tg-FDD model in comparison with WT mice (Fig. 7b, d). As impairing microglial TREM2 signaling reduces neuroinflammatory responses [34, 73, 74], we analyzed the expression of 30 inflammatory markers from the RNA-Seq data. No changes in the expression of interleukins were observed; however, expression of chemokines and TNF ligands such as Ccl27a, Cx3cl1, and C1qtnf4 was decreased in the Tg-FDD model in comparison with WT controls (Sup. Fig. 8). This may result from performing RNA-Seq on whole tissue, which may mask the unique inflammatory response of specific cell types. Since A1 astrocytes promote synaptic impairment [19, 75], we examined the expression of synaptic genes from the RNA-Seq data. IPA core analysis did not identify perturbed synaptic gene networks, suggesting no major synaptic impairment. Nevertheless, when we individually analyzed the expression levels of known pre- and postsynaptic markers, we observed a minor decrease in the expression of some postsynaptic markers such as PSD-95 and GABA _B_2 (Sup. Fig. 9). Overall, these data suggest that a decrease in TREM2 protein levels is associated with early-stage vascular amyloid pathology and early synaptic impairment in this CAA model.

**Fig. 8:**
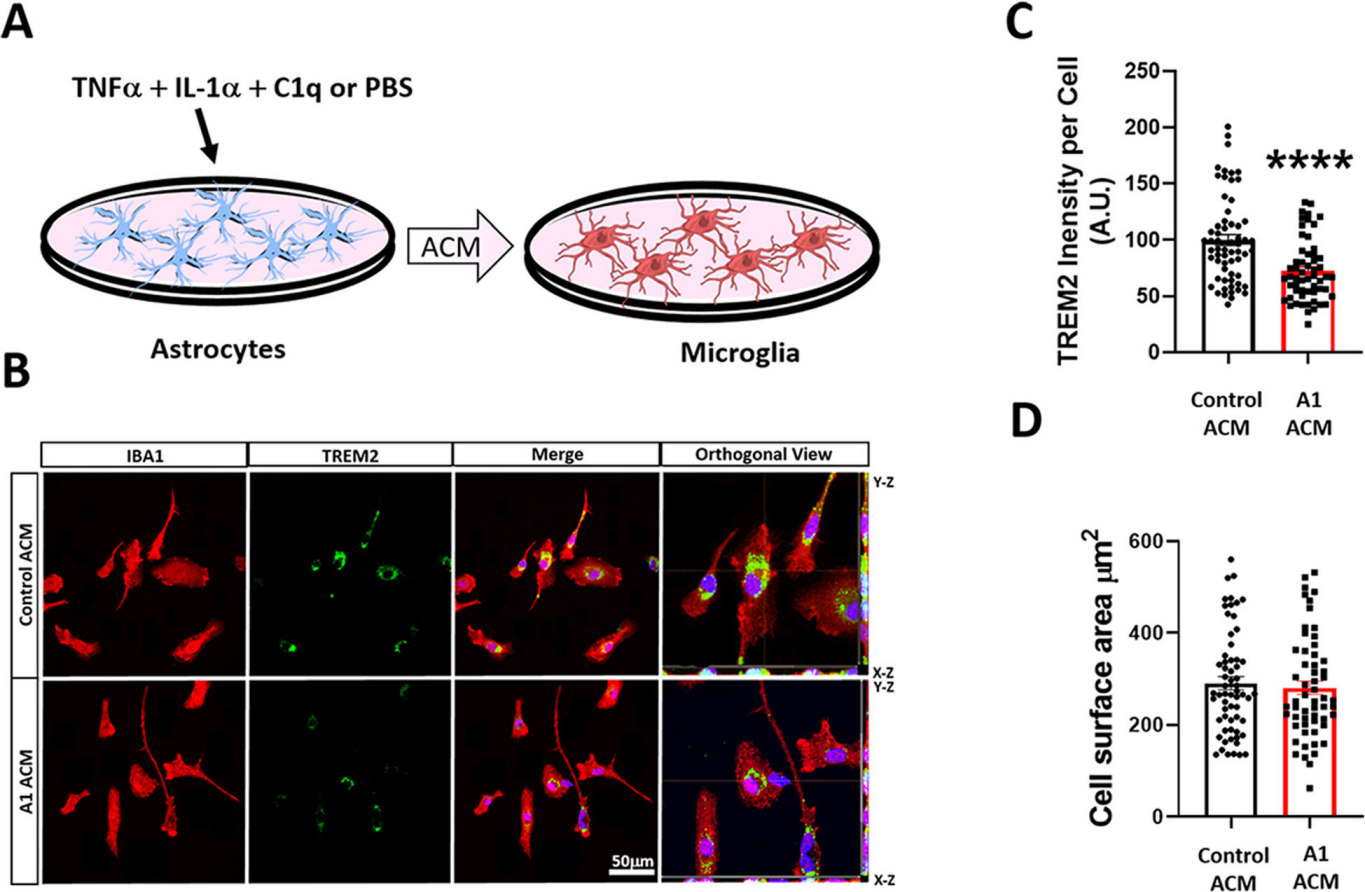
A1 astrocyte conditioned media decreases TREM2 protein in mouse primary microglia culture. **a** Schematic diagram showing A1 astrocytes induction by cytokine cocktail treatment (TNFα, IL-1α, and C1q) followed by microglia incubation with astrocyte conditioned media (ACM) from A1 astrocytes or control astrocytes solely treated with PBS. **b** Double immunofluorescence images of microglia (IBA1, red) and TREM2 (green) of primary WT mouse microglia culture treated with control or A1 ACM. Right panel shows orthogonal images of reconstructed three-dimensional views. c Intensity quantification of TREM2 per cell shows a decrease in TREM2 proteins in microglia incubated with A1 ACM. **d** Quantification of IBA1^+^ cell surface area shows no change in cell surface area between control and A1 ACM-treated groups. For quantifications, error bars represented ± SEM where eight to ten images were used for each experiment with *n* = 3 independent cultures and 20 cells per culture, where ****p < 0.0001 by unpaired Student’s *t* test. Scale bar 50 μm

Since our data suggest that astrogliosis occurs without significant microgliosis at the early stages of CAA pathology, we hypothesize that reactive A1 astrocytes could affect TREM2 protein levels in microglia. To investigate the ability of A1 astrocytes to modulate TREM2 protein levels in microglia, primary astrocyte cultures were treated with an A1-inducing cocktail of IL-1α, TNFα, and C1q or PBS (Fig. 8a). Then, primary cultures of microglia were treated with conditioned media from A1 astrocytes (A1-ACM) or untreated astrocytes control (ACM). Immunofluorescence analysis demonstrated that, in primary microglia incubated with A1-ACM, TREM2 protein levels were decreased in comparison with microglia treated with control ACM (Fig. 8b, c). Interestingly, no significant microglial activation was observed in cultures treated with A1-ACM (Fig. 8d). These results indicate that A1 astrocytes may affect TREM2 protein levels without inducing major microglial activation.

## Discussion

This study demonstrates that early pathology in a mouse model of CAA is associated with severe astrogliosis with an A1 astrocytic phenotype without significant microgliosis. Additionally, the immune response and lipid processing network are dysregulated in the Tg-FDD model. Further, validation demonstrated an impairment of cholesterol, ApoE, and TREM2 in this CAA model, as has been broadly demonstrated in AD mouse models, characterized by the accumulation of parenchymal amyloid plaques. Finally, A1 astrocytes induce a decrease in TREM2 protein levels in microglial culture.

The role of glial cells in the pathogenesis of several neurodegenerative diseases, including AD, has been a major research focus in recent years [10–16]. Since astrocytes and microglia activation has been described in CAA patients [29, 76], we studied the early glial response associated with early CAA pathology in Tg-FDD mice. Here, we report that vascular amyloid deposition in 9-month-old Tg-FDD animals contributes to robust immunoreactive and morphometric changes in astrocytes with increased glial branches and junctions in early CAA. Interestingly, astrocytic alterations associated with a premature vascular amyloid accumulation have also been reported in transgenic artic β-amyloid (acrAβ) mice [77], suggesting that severe astrocytic reactivity in early CAA is independent of amyloid species. In response to early vascular amyloid accumulation, microglial reactivity is unchanged, with no CD11B or IBA1 immunoreactive or morphometric changes in glial branches or junctions. Traditionally, studies of AD mouse models with parenchymal amyloid accumulation report the presence of both activated astrocytes and microglia [78–81]; however, our findings demonstrated that in a mouse model of CAA robust astrogliosis independent of microglial immune activation is a primary response to vascular amyloid deposition. The early astrocytic response in CAA could be due to the central role of astrocytes in the neurovascular unit in maintaining BBB integrity and cerebral blood flow regulation in physiological and pathological conditions [82–84]. For instance, changes in vascular components due to CNS injury have a direct influence on astrocyte signaling [85]. Astrocytes respond to and influence immune and inflammatory responses following insults such as excitotoxicity, ischemia, apoptosis, necrosis, and inflammatory cues by undergoing a pronounced transformative state called “reactive astrogliosis”, regulating inflammatory responses that can be either neuroprotective or neurotoxic and participating in migratory, phagocytic, and proteolytic activity [72]. The notion that the astrocyte and microglia phenotype observed in 9-month-old Tg-FDD results from a response to vascular amyloid deposits as opposed to overexpression of the mutant form of human BRI_2_ is supported by the fact that the transgene expression is under the control of the mouse prion promoter, which is exclusively expressed in neurons [37]. Therefore, no BRI_2_ is over-expressed in glial cells. The role of CAA in glial response in this model is also supported by the observation that no astrogliosis is detected in 3-month-old Tg-FDD mice, an age where no vascular amyloid deposits are observed [37].

It has recently been shown that activated microglia may directly polarize A1 astrocytes toward a neurotoxic phenotype [19]. Induced by a cytokine cocktail composed by TNFα, IL-1α, and C1q, A1 astrocytes can be identified by their upregulation of C3 and are shown to lose many normal homeostatic functions, such as the promotion of neuronal survival, neurite outgrowth, and synapse formation. This suggests that A1s are either unable to maintain synapses or actively disassemble them by releasing multiple complement components that help drive synaptic degeneration. A1 astrocytes also exert a toxic gain of function by secreting soluble neurotoxin(s) that induce neuronal and oligodendrocyte death, supporting the notion that A1 astrocytes are involved in the development of neurodegenerative diseases [19, 86]. In this study, we report for the first time that A1 astrocytes are highly abundant in a mouse model of CAA. No activated microglia were observed at early stages of CAA pathology, suggesting that inflammatory signaling derived from another source may be responsible for, and sufficient, to induce A1 astrocytes in CAA. While it is unknown if damaged endothelial or vascular cells polarize astrocytes to an A1 type, each can produce pro-inflammatory cytokines under toxic stimulation, supporting the notion that these cells are capable of A1 astrocytic induction [87, 88]. Blocking A1 astrocyte conversion is neuroprotective in Parkinson’s disease models [21]; however, their abolishment accelerated prion disease course in a mouse model [22]. This suggests that the role of these astrocytes is disease-specific, and further analysis is required to determine their contribution to CAA pathogenesis.

Recently, genome-wide association studies (GWAS), whole-genome sequencing (WGS), and gene-expression network analyses have revealed gene networks and common and rare genetic variants that are associated with LOAD. The majority of these genes are involved in lipid processing, endocytosis, and innate immunity [55, 56, 89, 90]. Further validations of many of these risk factors have focused on their effect on parenchymal amyloid pathology, with their influence in vascular amyloid pathology largely understudied. Gene expression analyses revealed dysregulation of immune response networks associated with lipid processing in the Tg-FDD model. Our RNA-Seq results showed an upregulation of the rare AD-associated genetic factors ABCA7 and TREM2 [57, 58], an upregulation of lipoprotein lipase (LPL), known to be involved in AD susceptibility and pathogenesis [59, 60], and downregulation of CX3CL1, which has known roles in neuroprotection and neurotoxicity in the context of AD [61–64]. This suggests that many of the immune and lipid-related genes, identified as risk factors for AD, may have a preponderant involvement in CAA pathogenesis. In support of this, a recent population-based study tested the association of distinct neuropathological features of AD with risk loci identified in GWAS studies. Remarkably, the authors observed an association between the ABCA7 and TREM2 loci with CAA and capillary-Aβ, respectively [91], further demonstrating a role for immune-related AD risk factors in vascular amyloid deposition.

Multiple threads of evidence connect cholesterol with AD [92]. Epidemiological studies suggest a positive correlation between hypercholesterolemia and increased AD risk, though the exact impact and mechanisms involved remain largely unknown. Previous reports have shown cholesterol accumulation in senile plaques of AD patients and mouse models alike [66], suggesting that cholesterol could play a role in the formation of amyloid plaques, and that amyloid impairs cholesterol trafficking and homeostasis. Here, our findings show prominent cholesterol accumulation associated with vascular amyloid in the Tg-FDD. This indicates that the dysregulation of cholesterol redistribution in CAA could play an important role in disease progression.

ApoE, predominantly produced by astrocytes, has been widely confirmed to regulate the redistribution and homeostasis of cholesterol within the brain and to affect the accumulation and clearance of Aβ-amyloid [70, 93, 94]. Here, we observed an association between ApoE and vascular amyloid deposits in Tg-FDD mice, possibly decreasing its solubility. These observations are supported by previous studies implicating ApoE as the principal component of parenchymal amyloid plaques and CAA [95, 96] and the major risk factor for both AD [97] and CAA [98]. The ApoE_4_ isoform is associated with total and vascular Aβ-amyloid levels [99], and the ApoE_2_ isoform is a risk factor for hemorrhagic CAA [97, 98]. Conversely, the ApoE_2_ isoform is protective in AD [100], suggesting that the mechanistic basis of ApoE-amyloid association differs between AD and CAA. The different influence of ApoE on the development of parenchymal plaques versus CAA has also been observed in mouse models. For instance, the over-expression of human ApoE_4_ in the APP^swe^ mouse model, characterized by the accumulation of amyloid plaques, results in a shift in the amyloid deposition from parenchyma to the vasculature [101]. Also, it was recently shown how in the 5XFAD model expressing murine ApoE and human ApoE_4_ parenchymal plaques colocalized with much more murine ApoE while vascular amyloid deposits contained more human ApoE_4_, suggesting that the type of ApoE dictates whether ApoE will lead to greater parenchymal plaques versus CAA [102]. Additional studies will be required to determine if ApoE impairment in the context of vascular amyloid originates from astrocytes or microglia. Pericytes may also be involved as a recent study demonstrated that ApoE plays a critical role in these vascular cells in the context of CAA [103].

The role of TREM2 in AD has become increasingly relevant as human variants of TREM2 have been associated with a 2- to -3-fold increased risk for developing LOAD [104]. Several cellular functions have been ascribed to TREM2, including the regulation of phagocytosis, inhibition of inflammatory signaling, promotion of cell survival, and cholesterol efflux [105, 106]. New studies have demonstrated that several apolipoproteins, including ApoE, are TREM2 ligands, and that the APOE-TREM2 pathway plays a fundamental role in the microglial homeostatic signature [15, 107]. However, the majority of TREM2 studies in AD relate to parenchymal amyloid deposition, and the role of TREM2 in vascular amyloid deposition and CAA is largely unknown [31, 34, 72, 73, 105]. Here, we demonstrated an increase in TREM2 mRNA levels in the Tg-FDD model in comparison with WT controls. Recently, a novel study demonstrated an increase in TREM2 mRNA levels in a rat model for CAA [108], supporting our findings. Interestingly, our biochemical analysis revealed a significant decrease in TREM2 protein levels. It is well known that transcriptome changes do not always correlate with protein abundance [109]; nevertheless, this discrepancy between mRNA and protein levels suggests the presence of either a post-translational mechanism promoting TREM2 degradation, or a post-translational modification directly in the TREM2 protein that decreases its stability. This decrease in protein levels could trigger an increase in TREM2 mRNA expression as a compensatory mechanism. Noteworthily, despite the significant decrease in TREM2 protein levels, no changes in microglial immunoreactivity were observed, suggesting that the decrease of TREM2 could be an early response of microglial reactivity to vascular amyloid deposits. Similar observations were made in an APP/PS1 mouse model characterized by the accumulation of parenchymal amyloid plaques, where the authors reported an increase of TREM2 mRNA and a decrease of TREM2 protein [110]. It was suggested that the lack of TREM2 protein may reduce microglia activity, ultimately leading to neuroinflammation [110]. Collectively, these data indicate that TREM2-related pathways could have an influence on vascular amyloidosis. Further, studies will be necessary to dissect in detail the effect of TREM2 in CAA [104] and the effect that ApoE impairment has on TREM2 via its ligand function [107] or the recently identified TREM2-APOE regulatory pathway responsible for microglial phenotypic change [15].

The decrease in TREM2 protein levels without significant changes in microglial reactivity in early vascular amyloid deposits in the Tg-FDD model is an unexpected and unique finding. However, activation of microglia can result in phenotypic and functional diversity—detrimental or beneficial—depending on the activation conditions. This duality in microglia has been reported in detail, but complex mechanisms that regulate these diverse states of activation remain unclear [111, 112]. It has been suggested that different stages of microglial activation may also depend on signaling from another CNS cell type [113]. Therefore, based on our analysis of the Tg-FDD model, where a preponderant A1 astrocyte reactivity was observed at the early stages of vascular amyloid pathology, we theorized that A1 astrocytes may contribute to microglia activation and decreased TREM2 levels. Interestingly, we observed that treating primary microglia with conditioned A1-ACM media decreases TREM2 intensity without significantly affecting microglia morphology, demonstrating the ability of A1 astrocytes to influence microglial steady state. The concept of A1 astrocytes influencing microglial activation is particularly interesting as studies have mainly focused on the ability of reactive microglia to influence A1 astrocytic phenotypes in disease and injury [19]; however, this concept has not been thoroughly explored in the context of astrocytes influencing microglia reactivity. Only a handful of studies have suggested a direct effect of astrocytes on microglia [114]. For instance, a high level of S100ß production in astrocytes contributes to iNOS and NO production in microglia [115]. In another study, astrocytes were able to downregulate the antigen-presenting function of invading monocytes [116]. Furthermore, a new study demonstrated that exposure to conditioned media from oxygen-glucose-deprived astrocytes activated microglia-promoted neuronal dendritogenesis [113]. Overall, these studies support the notion that in the Tg-FDD model, A1 astrocytes could secrete factor(s) that affect TREM2 protein stability and subsequently microglial activity.

Identifying the signals or factors used by A1 astrocytes to alter TREM2 protein levels in microglia and the role that ApoE could play provides a means to potentially regulate the neuroinflammatory response associated with CAA pathogenesis and in other cerebrovascular diseases where astrocytic reactivity could precede microglial response.

## Conclusions

Our study demonstrates that initial glial response associated with early-stage CAA is characterized by the upregulation of A1 astrocytes without significant microglial reactivity. This glial response is distinct from AD, where reactive microglia influence the A1 astrocyte phenotype. Noteworthy, gene expression analysis revealed that several AD risk factors involved in immune response and lipid processing could also have a preponderant role in CAA. This study contributes to the increasing evidence that brain cholesterol metabolism, ApoE, and TREM2 signaling are major players in the pathogenesis of AD-related dementias, including CAA. Understanding the basis for possible differential effects of the glial response, ApoE, and TREM2 signaling on parenchymal plaques versus vascular amyloid deposits provides important insight into neuroimmune mechanisms further understanding of neurodegenerative processes associated with AD and CAA, respectively, and promote the development of novel therapeutics and prevention strategies.

## Supplementary information

**Additional file 1: Supplementary-Figure 1.** Early vascular amyloid deposition in a transgenic mouse model for Familial Danish Dementia (Tg-FDD). Thio-S staining of brain from 9-month-old Tg-FDD mice demonstrated the presence of vascular amyloid deposits in the cortex, hippocampus, and cerebellum. Scale bar 50 μm or 100 μm.

**Additional file 2: Supplementary-Figure 2.** No changes in microglia immunoreactivity is observed in Tg-FDD. Double immunofluorescence images of amyloid (Thio-S, green) and CD11B (red) in the cortex, hippocampus, and cerebellum of 9-month-old WT or Tg-FDD mice showed no differences in CD11B % area. Results are shown as the mean ± SEM of n = 3-4. No significant differences where observed by Unpaired Student's t test. Scale bar 100 μm.

**Additional file 3: Supplementary-Figure 3.** Reactive astrogliosis is accentuate in the perivascular regions in Tg-FDD mice. Triple immunofluorescence of astrocytes (GFAP, cyan), amyloid (Thio-S, green), and smooth muscle actin (SMA, red) in the cortex, hippocampus, and cerebellum of 9-month-old Tg-FDD mice and WT controls. Major presence of reactive astrogliosis is observed in the perivascular region of Thio-S positive vasculature in Tg-FDD mice. Scale bar 50 μm.

**Additional file 4: Supplementary-Figure 4.** No glial immunoreactive is observed in 3-month-old Tg-FDD mice. A) Double immunofluorescence images of amyloid (Thio-S, green) and astrocytes (GFAP, red) and quantification of GFAP^+^ area (%) in WT and Tg-FDD mice. **B)** Double immunofluorescence images of amyloid (Thio-S, green) and microglia (IBA1, red) and quantification of IBA1^+^ area (%) in WT and Tg-FDD mice. All are representative images of the brain regions of 3-month-old WT or Tg-FDD mice. Results are shown as the mean ± SEM of n = 3. No significant differences where observed by Unpaired Student's t test. Scale bar 100 μm.

**Additional file 5: Supplementary-Figure 5.** Cholesterol accumulation in Tg-FDD. The presence of cholesterol was detected by fluorescent microscopy images using Filipin (green). Cholesterol accumulation is not observed prior to CAA pathology in 3-month-old Tg-FDD or in 9-month-old WT animals but is observed at early stages of CAA deposition in 9-month-old Tg-FDD mice. Scale bar 100 μm.

**Additional file 6: Supplementary-Figure 6.** Reactive astrocytes cluster around cholesterol deposits in Tg-FDD mice. Double immunofluorescence of astrocytes (GFAP, red) and cholesterol (Filipin, green) in Tg-FDD. Three-dimensional view shows that astrocytes are surrounding cholesterol deposits. Scale bar 50 μm.

**Additional file 7: Supplementary-Figure 7.** Increased TREM2 mRNA levels in cerebral hemisphere and cerebellum in Tg-FDD mice. mRNA levels of TREM2 in the cerebral hemisphere and cerebellum of 9-month-old WT and Tg-FDD mice were measured by quantitative reverse transcription-PCR (qRT-PCR). Data was normalized to the levels of GAPDH mRNA. Relative quantitation was performed using 2^-ΔΔCt^ (fold change) method. Results are shown as the mean ± SEM of n = 3. Asterisks indicate significant differences, where * *p* < 0.05 and ** *p* < 0.01.

**Additional file 8: Supplementary-Figure 8.** No major changes are observed in inflammatory and chemotactic markers in Tg-FDD mice. **A)** RNA-Seq data from 9-month-old Tg-FDD and WT mice showed no difference in expression of interleukins such as IL-1a, IL 15, IL33, IL 22, IL 16, IL 34, IL12a, IL 18, and IL 11. **B)** The only tumor necrosis factor ligand that statistically decreased in Tg-FDD was C1qtnf4. **C)** No major changes in chemokines expression were observed in Tg-FDD mice. Only Ccl27a and CX3CL1 were statistically decreased. Results are shown as the mean ± SEM of n = 3. Asterisks indicate significant differences, where **** *p* < 0.0001. RPKM = Reads Per Kilobase of transcript, per Million mapped reads.

**Additional file 9: Supplementary-Figure 9.** Synaptic markers expression in Tg-FDD mice. RNA-Seq data from 9-month-old Tg-FDD and WT mice showed no difference in presynaptic markers synaptophysin (Syp), Synapsin 1 (Syn1), Synapsin 2 (Syn2), and Synapsin 3 (Syn3). No changes were observed in the postsynaptic markers GLUR1 and GluN1. The postsynaptic markers PSD95 and GABA _B_2 were statistically decreased in Tg-FDD in comparison with WT mice. Results are shown as the mean ± SEM of n = 3. Asterisks indicate significant differences, where * *p* < 0.05 and ** *p* < 0.01. RPKM = Reads Per Kilobase of transcript, per Million mapped reads.

## Data Availability

The RNA-Seq data supporting the conclusions of this article are available in the Gene Expression Omnibus (GEO): GSE150394.

## Abbreviations

Aβ: Amyloid-beta protein
ABCA7: ATP-binding cassette sub-family A member 7
AD: Alzheimer’s disease
ADan: Danish amyloid
C1q: Complement component 1q
C3: Complement component 3
CAA: Cerebral amyloid angiopathy
CX3CL1: CX3C chemokine ligand 1
GFAP: Glial fibrillary acidic protein
Iba-1: Ionized calcium-binding adapter molecule 1
IL-1α: Interleukin 1 alpha
LOAD: Late-onset Alzheimer’s disease
LPL: Lipoprotein lipase
RT-qPCR: Quantitative reverse transcription PCR
SD: Standard deviation
Tg-FDD: Transgenic mice that express Danish amyloid in the brain
Thio-S: Thioflavin S
TNFα: Tumor necrosis factor alpha
TREM2: Triggering receptors expressed on myeloid cells 2
