# Supplementary figures and images for: A1 reactive astrocytes and a loss of TREM2 are associated with an early stage of pathology in a mouse model of cerebral amyloid angiopathy
Source: J Neuroinflammation. 2020 Jul 25;17:223. doi: 10.1186/s12974-020-01900-7 (PMC7382050; doi:10.1186/s12974-020-01900-7)

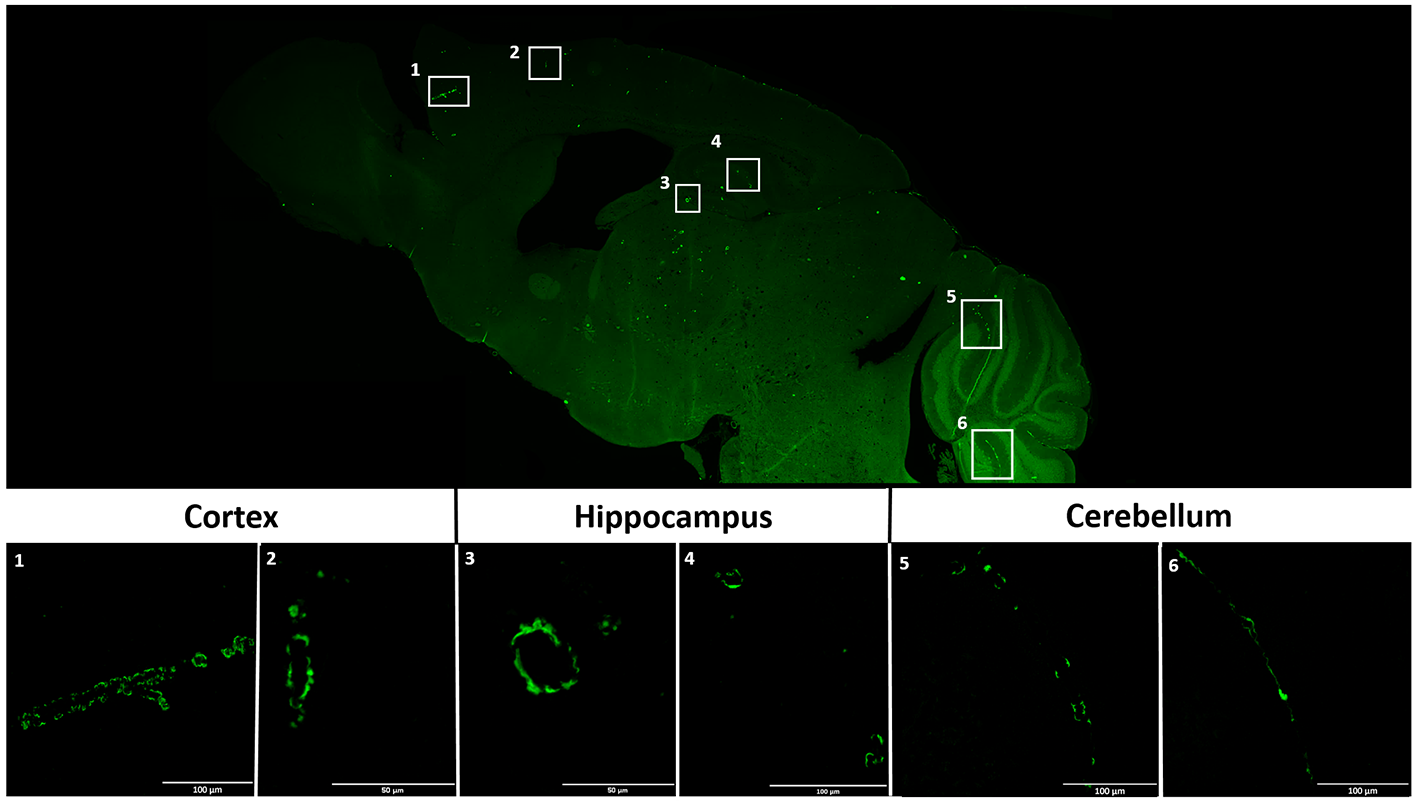

Supplement: Supplementary file 1 — Additional file 1: Supplementary-Figure 1. Early vascular amyloid deposition in a transgenic mouse model for Familial Danish Dementia (Tg-FDD). Thio-S staining of brain from 9-month-old Tg-FDD mice demonstrated the presence of vascular amyloid deposits in the cortex, hippocampus, and cerebellum. Scale bar 50 μm or 100 μm. [file 12974_2020_1900_MOESM1_ESM.tif]

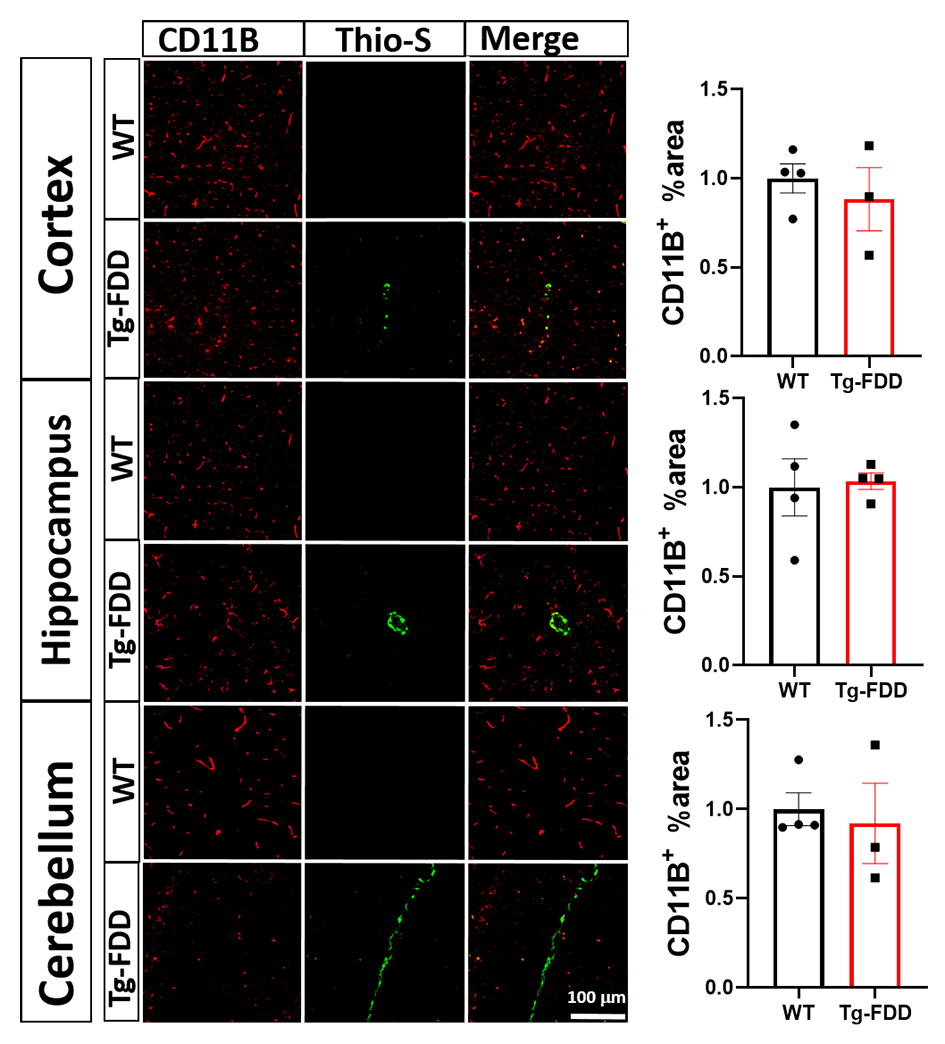

Supplement: Supplementary file 2 — Additional file 2: Supplementary-Figure 2. No changes in microglia immunoreactivity is observed in Tg-FDD. Double immunofluorescence images of amyloid (Thio-S, green) and CD11B (red) in the cortex, hippocampus, and cerebellum of 9-month-old WT or Tg-FDD mice showed no differences in CD11B % area. Results are shown as the mean ± SEM of n = 3-4. No significant differences where observed by Unpaired Student's t test. Scale bar 100 μm. [file 12974_2020_1900_MOESM2_ESM.tif]

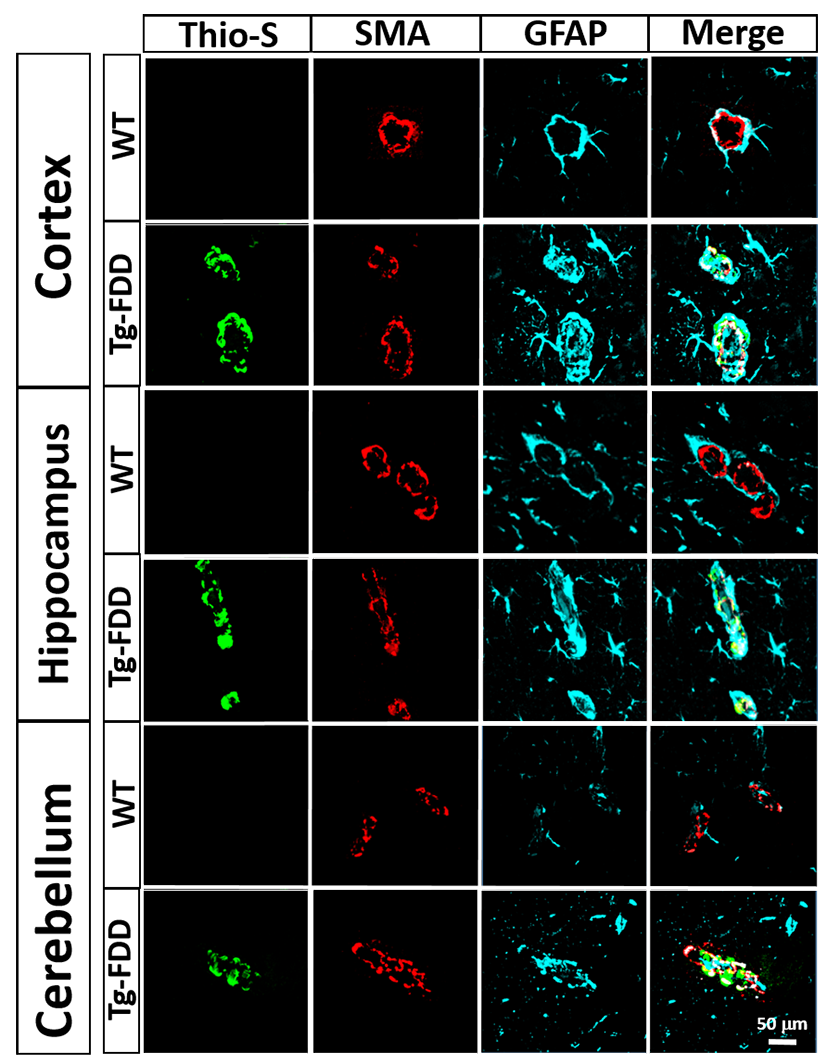

Supplement: Supplementary file 3 — Additional file 3: Supplementary-Figure 3. Reactive astrogliosis is accentuate in the perivascular regions in Tg-FDD mice. Triple immunofluorescence of astrocytes (GFAP, cyan), amyloid (Thio-S, green), and smooth muscle actin (SMA, red) in the cortex, hippocampus, and cerebellum of 9-month-old Tg-FDD mice and WT controls. Major presence of reactive astrogliosis is observed in the perivascular region of Thio-S positive vasculature in Tg-FDD mice. Scale bar 50 μm. [file 12974_2020_1900_MOESM3_ESM.tif]

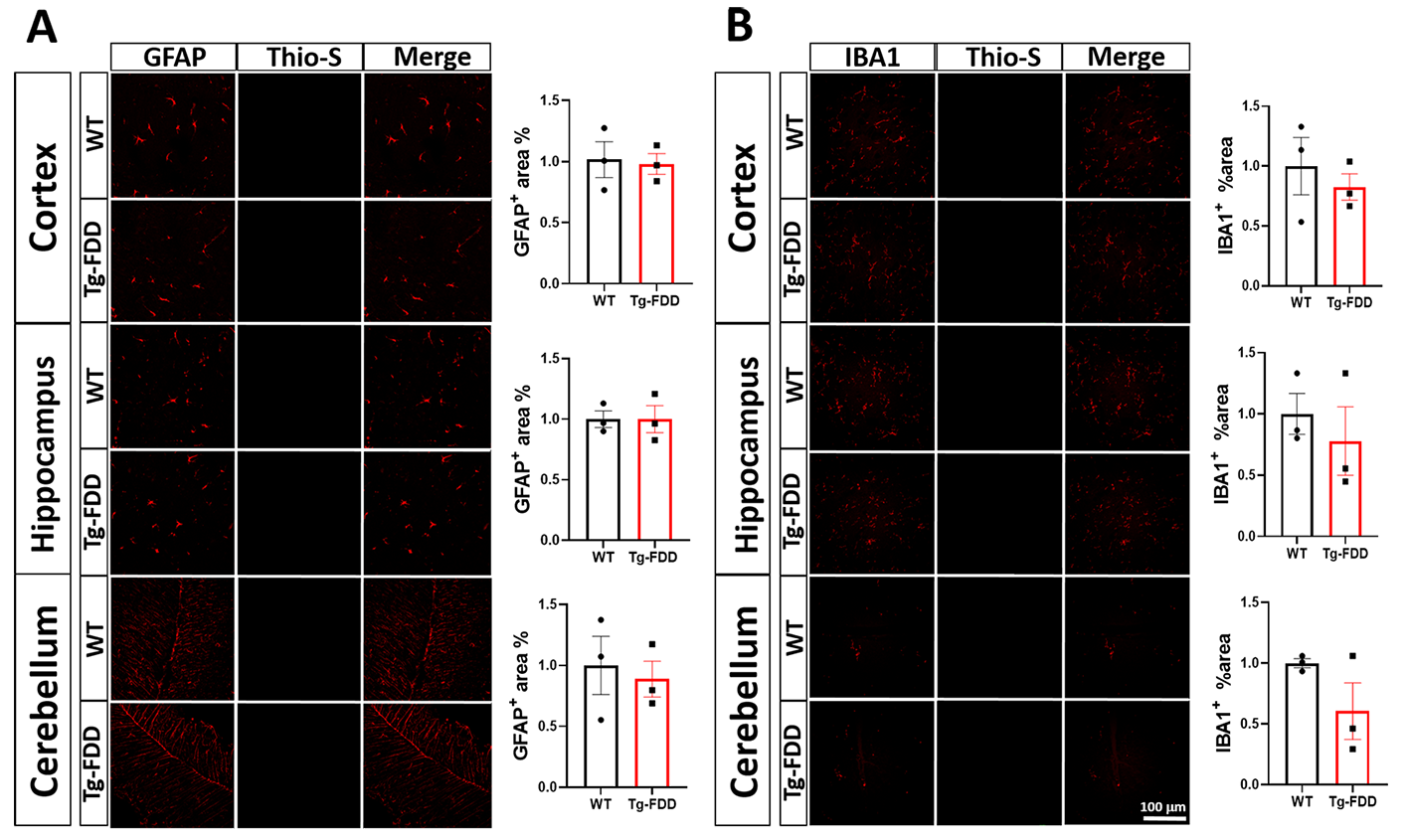

Supplement: Supplementary file 4 — Additional file 4: Supplementary-Figure 4. No glial immunoreactive is observed in 3-month-old Tg-FDD mice. A) Double immunofluorescence images of amyloid (Thio-S, green) and astrocytes (GFAP, red) and quantification of GFAP+ area (%) in WT and Tg-FDD mice. B) Double immunofluorescence images of amyloid (Thio-S, green) and microglia (IBA1, red) and quantification of IBA1+ area (%) in WT and Tg-FDD mice. All are representative images of the brain regions of 3-month-old WT or Tg-FDD mice. Results are shown as the mean ± SEM of n = 3. No significant differences where observed by Unpaired Student's t test. Scale bar 100 μm. [file 12974_2020_1900_MOESM4_ESM.tif]

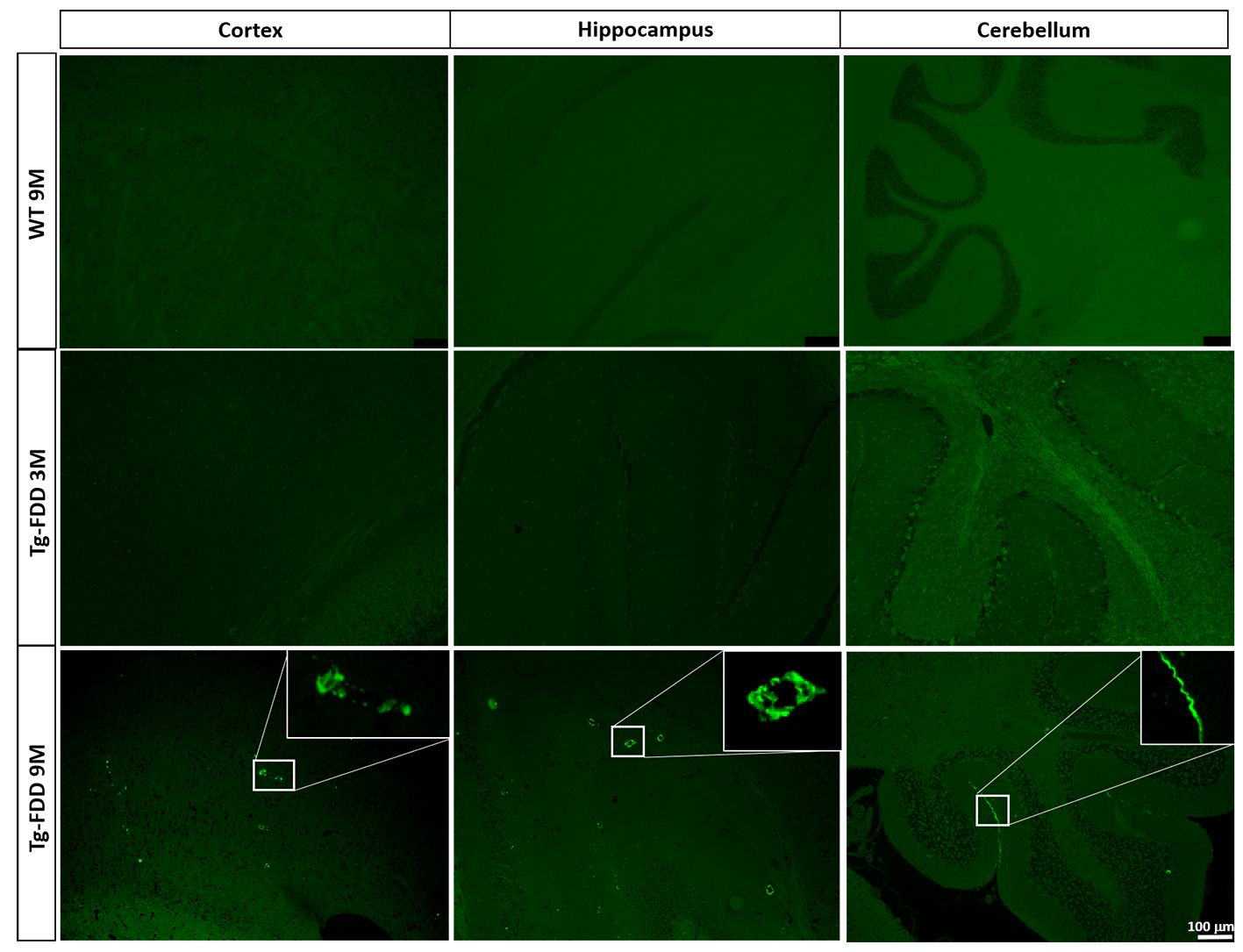

Supplement: Supplementary file 5 — Additional file 5: Supplementary-Figure 5. Cholesterol accumulation in Tg-FDD. The presence of cholesterol was detected by fluorescent microscopy images using Filipin (green). Cholesterol accumulation is not observed prior to CAA pathology in 3-month-old Tg-FDD or in 9-month-old WT animals but is observed at early stages of CAA deposition in 9-month-old Tg-FDD mice. Scale bar 100 μm. [file 12974_2020_1900_MOESM5_ESM.tif]

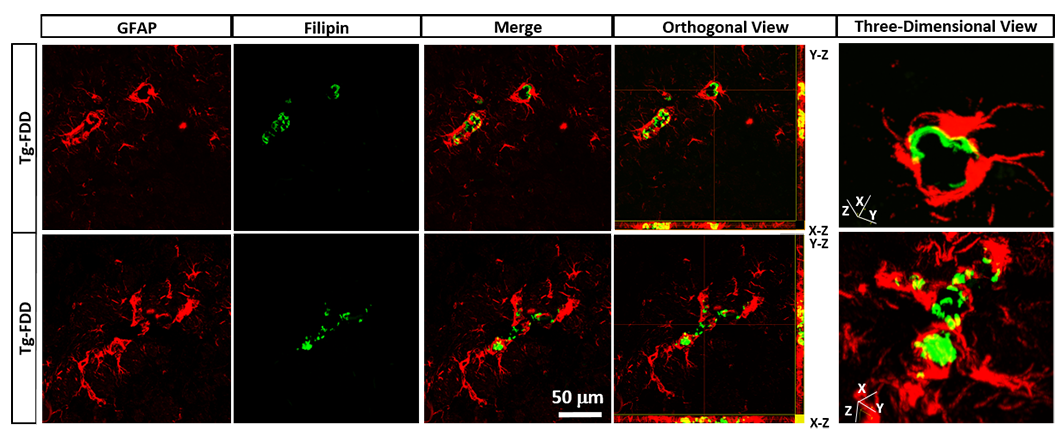

Supplement: Supplementary file 6 — Additional file 6: Supplementary-Figure 6. Reactive astrocytes cluster around cholesterol deposits in Tg-FDD mice. Double immunofluorescence of astrocytes (GFAP, red) and cholesterol (Filipin, green) in Tg-FDD. Three-dimensional view shows that astrocytes are surrounding cholesterol deposits. Scale bar 50 μm. [file 12974_2020_1900_MOESM6_ESM.tif]

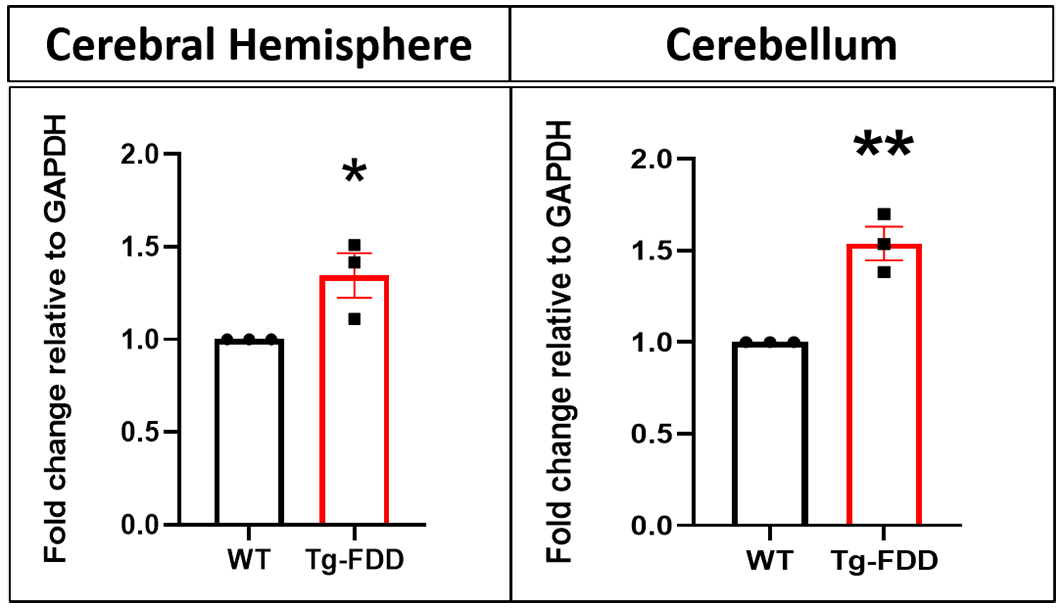

Supplement: Supplementary file 7 — Additional file 7: Supplementary-Figure 7. Increased TREM2 mRNA levels in cerebral hemisphere and cerebellum in Tg-FDD mice. mRNA levels of TREM2 in the cerebral hemisphere and cerebellum of 9-month-old WT and Tg-FDD mice were measured by quantitative reverse transcription-PCR (qRT-PCR). Data was normalized to the levels of GAPDH mRNA. Relative quantitation was performed using 2-ΔΔCt (fold change) method. Results are shown as the mean ± SEM of n = 3. Asterisks indicate significant differences, where * p < 0.05 and ** p < 0.01. [file 12974_2020_1900_MOESM7_ESM.tif]

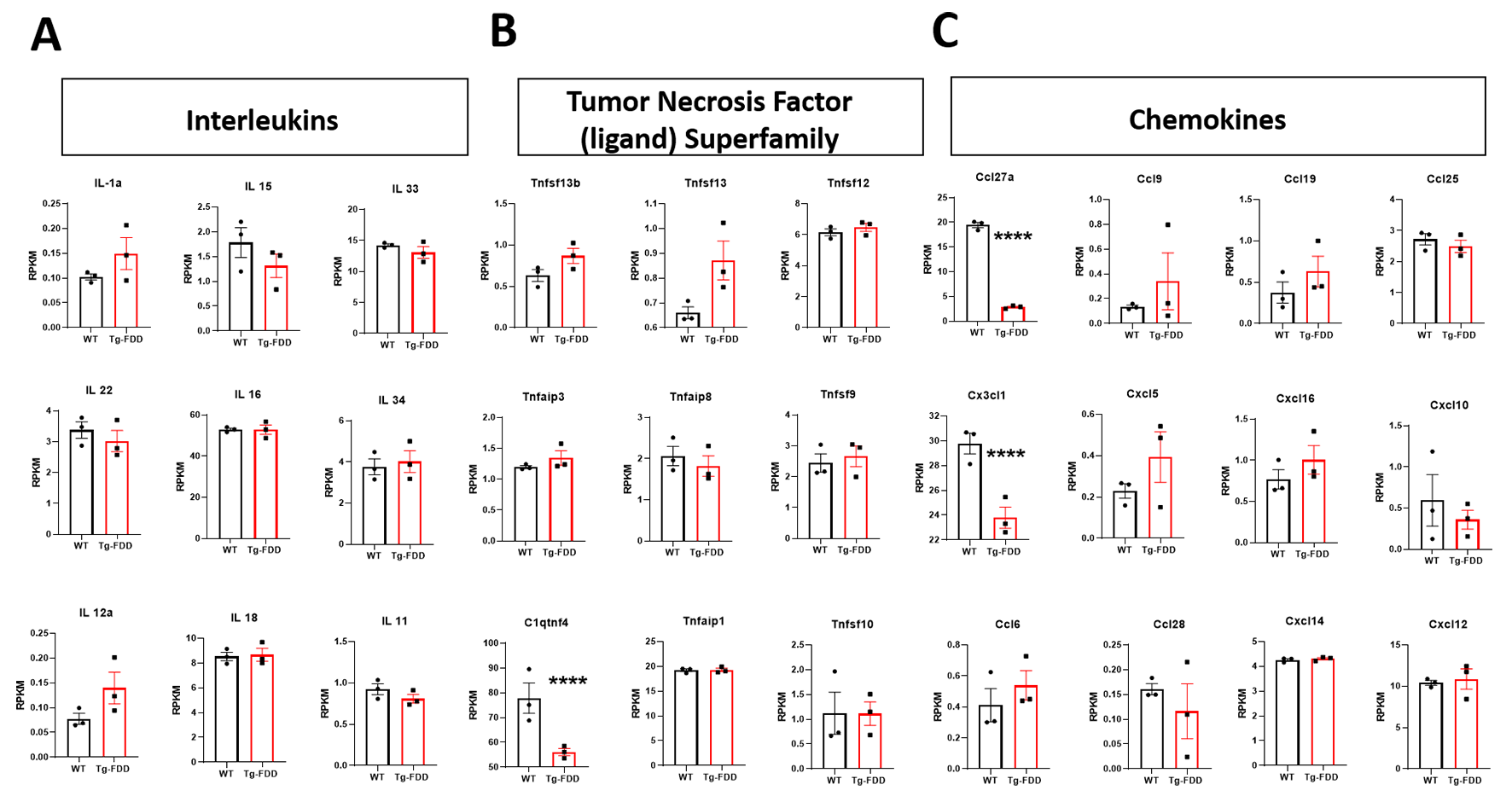

Supplement: Supplementary file 8 — Additional file 8: Supplementary-Figure 8. No major changes are observed in inflammatory and chemotactic markers in Tg-FDD mice. A) RNA-Seq data from 9-month-old Tg-FDD and WT mice showed no difference in expression of interleukins such as IL-1a, IL 15, IL33, IL 22, IL 16, IL 34, IL12a, IL 18, and IL 11. B) The only tumor necrosis factor ligand that statistically decreased in Tg-FDD was C1qtnf4. C) No major changes in chemokines expression were observed in Tg-FDD mice. Only Ccl27a and CX3CL1 were statistically decreased. Results are shown as the mean ± SEM of n = 3. Asterisks indicate significant differences, where **** p < 0.0001. RPKM = Reads Per Kilobase of transcript, per Million mapped reads. [file 12974_2020_1900_MOESM8_ESM.tif]

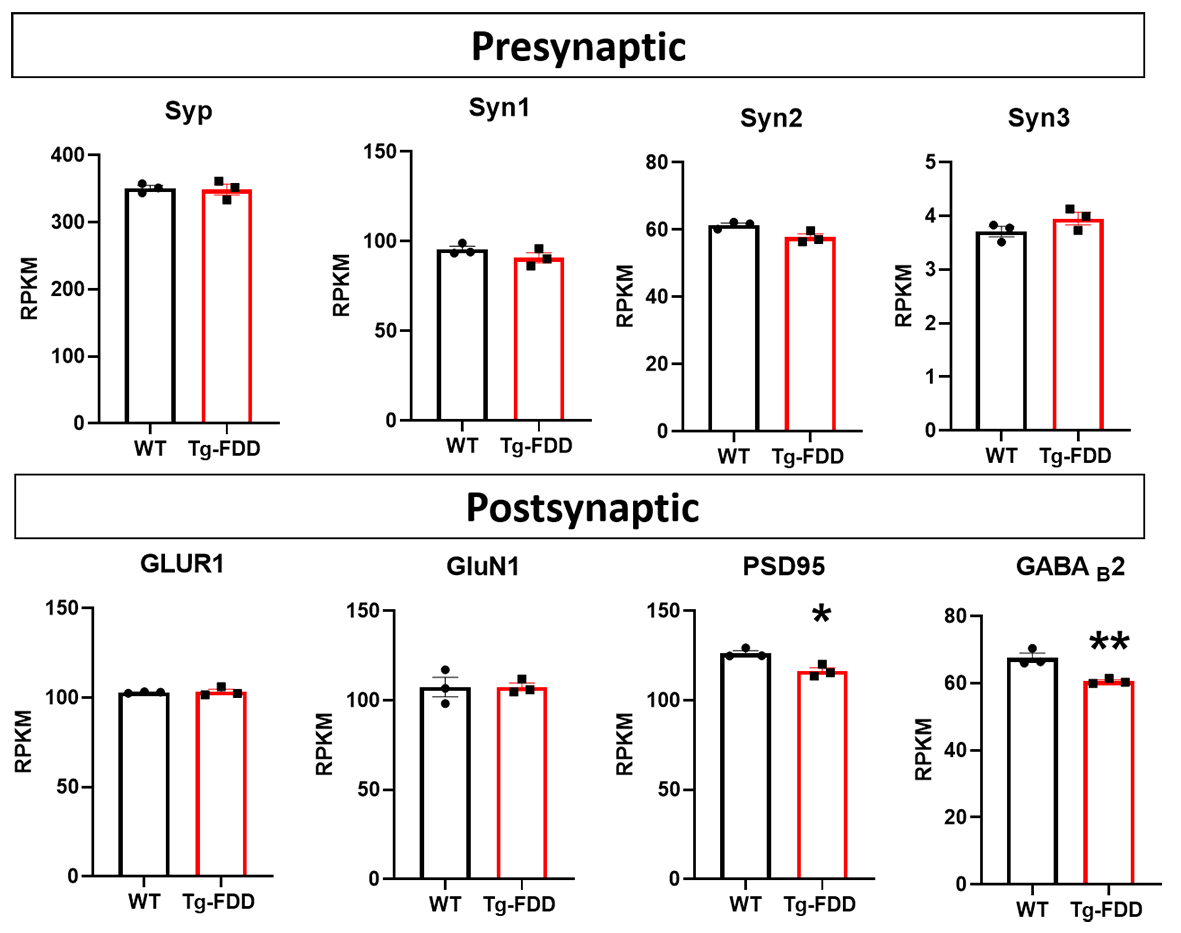

Supplement: Supplementary file 9 — Additional file 9: Supplementary-Figure 9. Synaptic markers expression in Tg-FDD mice. RNA-Seq data from 9-month-old Tg-FDD and WT mice showed no difference in presynaptic markers synaptophysin (Syp), Synapsin 1 (Syn1), Synapsin 2 (Syn2), and Synapsin 3 (Syn3). No changes were observed in the postsynaptic markers GLUR1 and GluN1. The postsynaptic markers PSD95 and GABA B2 were statistically decreased in Tg-FDD in comparison with WT mice. Results are shown as the mean ± SEM of n = 3. Asterisks indicate significant differences, where * p < 0.05 and ** p < 0.01. RPKM = Reads Per Kilobase of transcript, per Million mapped reads. [file 12974_2020_1900_MOESM9_ESM.tif]
